# The dynamics of the female microbiome: unveiling abrupt changes of microbial domains across body sites from prepartum to postpartum phases

**DOI:** 10.1128/spectrum.00147-24

**Published:** 2024-06-25

**Authors:** Charlotte J. Neumann, Manuela-Raluca Pausan, Victoria Haid, Eva-Christine Weiss, Vassiliki Kolovetsiou-Kreiner, Bettina Amtmann, Petra Winkler, Alexander Mahnert, Evelyn Jantscher-Krenn, Christine Moissl-Eichinger

**Affiliations:** 1 Diagnostic and Research Institute of Hygiene, Microbiology and Environmental Medicine, Medical University of Graz, Graz, Styria, Austria; 2 Department of Obstetrics and Gynecology, Medical University of Graz, Graz, Styria, Austria; 3 Research Unit Early Life Determinants (ELiD), Medical University of Graz, Graz, Styria, Austria; 4 BioTechMed, Graz, Styria, Austria; Wayne State University, Detroit, Michigan, USA

**Keywords:** pregnancy, perinatal, microbiome, archaea, fungi, amplicon sequencing, metabolomics, urine, vaginal, oral

## Abstract

The microbial ecosystem of women undergoes enormous changes during pregnancy and the perinatal period. Little is known about the extent of changes in the maternal microbiome beyond the vaginal cavity and its recovery after birth. In this study, we followed pregnant women [maternal prepartum (mpre), *n* = 30] into the postpartum period [1 month postpartum, maternal postpartum (mpost), *n* = 30]. We profiled their oral, urinary, and vaginal microbiome; archaeome; mycobiome; and urinary metabolome and compared them with those of nonpregnant (np) women (*n* = 29). Overall, pregnancy status (np, mpre, and mpost) had a smaller effect on the microbiomes than body site, but massive transitions were observed for the oral and urogenital (vaginal and urinary) microbiomes. While the oral microbiome fluctuates during pregnancy but stabilizes rapidly within the first month postpartum, the urogenital microbiome is characterized by a major remodeling caused by a massive loss of *Lactobacillus* and thus a shift from Vaginal Community State Type (CST) I (40% of women) to CST IV (85% of women). The urinary metabolome rapidly reached an np-like composition after delivery, apart from lactose and oxaloacetic acid, which were elevated during active lactation. Fungal and archaeal profiles were indicative of pregnancy status. *Methanobacterium* signatures were found mainly in np women, and *Methanobrevibacter* showed an opposite behavior in the oral cavity (increased) and vagina (decreased) during pregnancy. Our findings suggest that the massive remodeling of the maternal microbiome and metabolome needs more attention and that potential interventions could be envisioned to optimize recovery and avoid long-term effects on maternal health and subsequent pregnancies.

**IMPORTANCE:**

The perinatal microbiome is of specific interest for the health of the mother and infant. We therefore investigate the dynamics of the female microbiome from nonpregnant over prepartum to the postpartum period in urine and the oral and vaginal cavities. A specific focus of this study is put not only on the bacterial part of the microbiome but also on the underinvestigated contribution of fungi and archaea. To our knowledge, we present the first study highlighting those aspects. Our findings suggest that the massive remodeling of the maternal microbiome and metabolome needs more attention and that potential interventions could be envisioned to optimize recovery and avoid long-term effects on maternal health and subsequent pregnancies.

## INTRODUCTION

The female body undergoes profound changes from conception to pregnancy and the perinatal period that affect hormonal status, metabolism, the immune system, and the microbiome (1). For example, the maternal microbiome changes during pregnancy, a period characterized by low-grade inflammation, increased fat storage, and insulin resistance (2). However, knowledge of the transition of the microbiome from nonpregnant to pregnant and from prepartum to postpartum is relatively sparse, particularly with respect to bacteriomes, mycobiomes, and archaeomes, and beyond vaginal and gastrointestinal body sites.

It is well known that the vaginal microbiome is altered by pregnancy. In general, a healthy vaginal microbiome is predominated by lactobacilli, which are thought to maintain low pH and produce bacteriocins that inhibit pathogen growth (3). During pregnancy, vaginal diversity decreases and the predominance of *Lactobacillus*-species increases (4), probably to protect the mother and fetus from infection (5). This rise in *Lactobacillus* may be due to increased estrogen levels during pregnancy, which causes maturation of the vaginal epithelium and consequent accumulation of glycogen. Degraded by the host, products such as maltose, maltotriose, and maltotetraose then promote the growth of lactic acid bacteria (6, 7).

Pregnancy also affects the oral and urinary microbiome. During pregnancy, the oral microbiome is of particular clinical interest, as many women suffer from bleeding gums, gingivitis, or periodontitis (8, 9), which are likely caused by microbial disturbances associated with immune modulation and hormonal changes (10). Gingival diseases are associated with adverse pregnancy outcomes (11). Similarly, the urinary microbiome of pregnant women is clinically relevant because urinary tract infections increase the risk of preterm birth (12). A previous study showed that certain microbial taxa in urine, namely, *Ureaplasma urealyticum*, were associated with preterm birth even in the absence of urinary tract infections (13).

Parturition marks a rather abrupt change for both the child and the mother, which also brings dramatic changes in microbial niches. For the newborn, birth is a starting point for microbial colonization. The transfer of the microbiome from the mother to the child plays an essential role in the development of the infant’s microbiome and immune system. For the mother, childbirth is a turning point that also affects her microbiome transitioning from a pregnant to a postpartum state. In addition, the immediate postpartum period is a difficult time, characterized, for example, by drastic hormonal changes, increased energy and nutrient demands (also due to breastfeeding), sleep disturbances, or depressive symptoms (14, 15), which can affect the microbiome and *vice versa* (15).

After delivery, the vaginal microbial community shifts from a *Lactobacillus-*predominated microbiome to a *Lactobacillus*-depleted microbiome (15), likely caused by the rapid decline in estrogen levels (3). This phase is associated with diseases such as bacterial vaginosis and vulvovaginal candidiasis (16, 17). Cultivation-based studies have shown that *Gardnerella*, *Peptococcus*, *Bacteroides*, *Staphylococcus*, *Streptococcus*, and *Ureaplasma* species (3) are increased, which is associated with postpartum endometritis, which occurs in 1%–3% of all deliveries (18). It is estimated that only about 50% of women achieve a healthy *Lactobacillus*-predominated status at 1 year postpartum because *Lactobacillus crispatus* does not return to predominance (14).

Although awareness of the problematic recovery of the vaginal microbiome to the prepregnancy status quo has been raised, potentially impacting subsequent pregnancies (14), knowledge of the transition of the maternal microbiome of other body sites as well as the dynamics of the nonbacterial microbiome is still surprisingly sparse as already indicated elsewhere (19). Here, we highlight the transition of microbiomes in the oral and urogenital (urinal and vaginal) body sites of 30 women from the prepartum to 1 month postpartum and place it in context with the microbiomes of nonpregnant women. We use specific detection and analysis methods for the archaeome and mycobiome to provide a broader view of the holistic microbiome.

## RESULTS

### Study set-up and general description

The aim of the study was to understand the extent of imbalance of the bacterial and nonbacterial, oral, and urogenital (urinal and vaginal) microbiomes in postpartum women compared with their pregnant status or nonpregnant individuals. Thirty healthy pregnant participants were enrolled in this study to investigate the microbial changes with and after pregnancy. For this purpose, we recruited women in the third trimester (*n* = 30, “maternal prepartum” = “mpre”), who provided samples at this time and 1 month after delivery (*n* = 30, “maternal postpartum” = “mpost;” Cesarean section (CS) *n* = 15, vaginal delivery *n* = 15). The study also included nonpregnant female controls for comparison (*n* = 29, “nonpregnant” = “np”). The following samples were collected from all three study groups: oral swabs, noncathetered urine, and vaginal swabs. A total of 353 samples were processed by amplicon sequencing targeting the overall microbiome (“universal,” targeting both archaeal and bacterial, but mainly bacterial 16S rRNA genes), archaea, and fungi. Nuclear magnetic resonance spectroscopy (NMR)-based metabolomics was performed for the urine samples. An overview on the samples, number of reads, and Amplicon Sequence Variant (ASV) numbers is shown in Fig. S1 and S2. The characteristics of the cohorts are shown in Table 1.

**TABLE 1:** Characteristics of the study cohort, statistically compared between the mpre and mpost groups and the np group as well as within the pregnant group between those who delivered via CS versus those who delivered vaginally

Characteristics	mpre and mpost (*n* = 30)	np (*n* = 29)	*P* value
Mean age (yr) ± SD (range)	34.5 ± 4.7 (27–44)	32.0 ± 5.7 (19–43)	0.083^*a*^
Mean BMI prepregnancy (kg/m^2^) ± SD (range)	22.4 ± 4.1 (17.3–37.0)	22.6 ± 3.6 (17.2–31.5)	0.968^*b*^

Pregnant group			
Characteristics	CS (*n* = 15)	Vaginal birth (*n* = 15)	*P* value
Mean age (yr) ± SD (range)	35.8 ± 5.1 (28–44)	33.1 ± 3.9 (27–39)	0.113^*a*^
Mean BMI at sampling (kg/m^2^) ± SD (range)	27.3 ± 6.2 (21.8–47.6)	27.6 ± 3.4 (22.2–34.0)	0.436^*b*^
Mean BMI prepregnancy (kg/m^2^) ± SD (range)	22.2 ± 4.5 (18.3–37.0)	23.0 ± 3.8 (17.3–29.4)	0.285^*b*^
Pregnancy days	272.7 ± 7.3 (258–286)	283.1 ± 3.5 (276–289)	<0.001^*a*^
Infant weight (g)	3408.6 ± 465.5 (2,510–4,250)	3486.0 ± 269.8 (3,040–4,110)	0.582^*a*^
Days in hospital	3.9 ± 0.7 (2–5)	3.6 ± 1.5 (1–6)	0.87^*b*^
Sex of the infant	F: 8; M: 7	F: 6; M: 9	0.715^*c*^

^*a*^ *t*-test.

^*b*^ Mann-Whitney U test (independent samples).

^*c*^ Fisher's exact *t*-test

### Bacterial and fungal microbiomes are affected by pregnancy status

**Fig 1:**
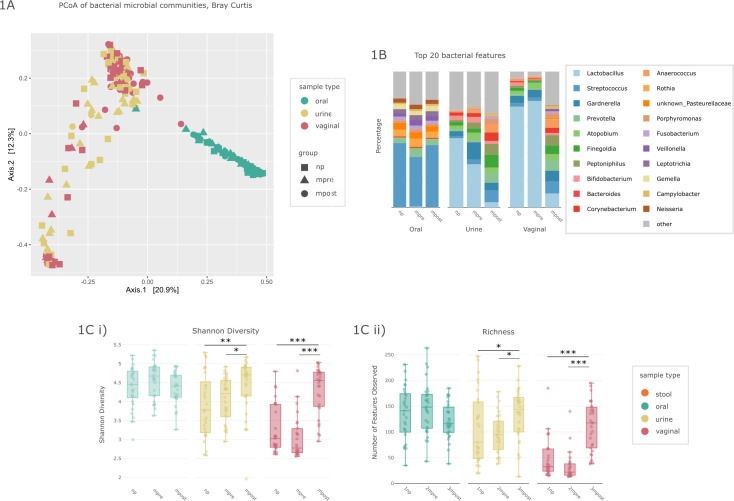
Beta diversity, alpha diversity, and composition of the bacterial microbiome. (**A**) Principal Coordinate Analysis (PCoA) of sample type (color) and group (shape) with Bray-Curtis distance matrix on ASV level, PERMANOVA. (**B**) Composition of the top 20 bacterial genera in relative abundance, depicted by sample type and group. (**C**) Alpha diversity of sample types by group on ASV level of (i) Shannon diversity and (ii) richness. Significance levels are indicated with asterisks for ***P  <  0.001, **P  <  0.01, and *P  <  0.05, Kruskal-Wallis test, adjusted with Bonferroni.

Figure 1 shows that the similarity of the bacterial microbiomes of all samples could be mainly explained by the body site they are taken from (PERMANOVA, Bray-Curtis, *P* = 0.001, *R*^2^ = 0.194) and, with a smaller effect size, also by perinatal status (np, mpre, and mpost; PERMANOVA, Bray-Curtis, *P* = 0.001, *R*^2^ = 0.03). *Lactobacillus* was the most prominent genus, found primarily in vaginal and urinary np and mpre samples, followed by *Streptococcus* (mostly oral samples) (Fig. 1B).

### Methanogenic archaea: indicators for pregnancy?

Archaeal signatures were analyzed semi-quantitatively (“universal approach”) alongside bacteria and qualitatively in a separate “nested approach” specifically targeting archaea.

**Fig 2:**
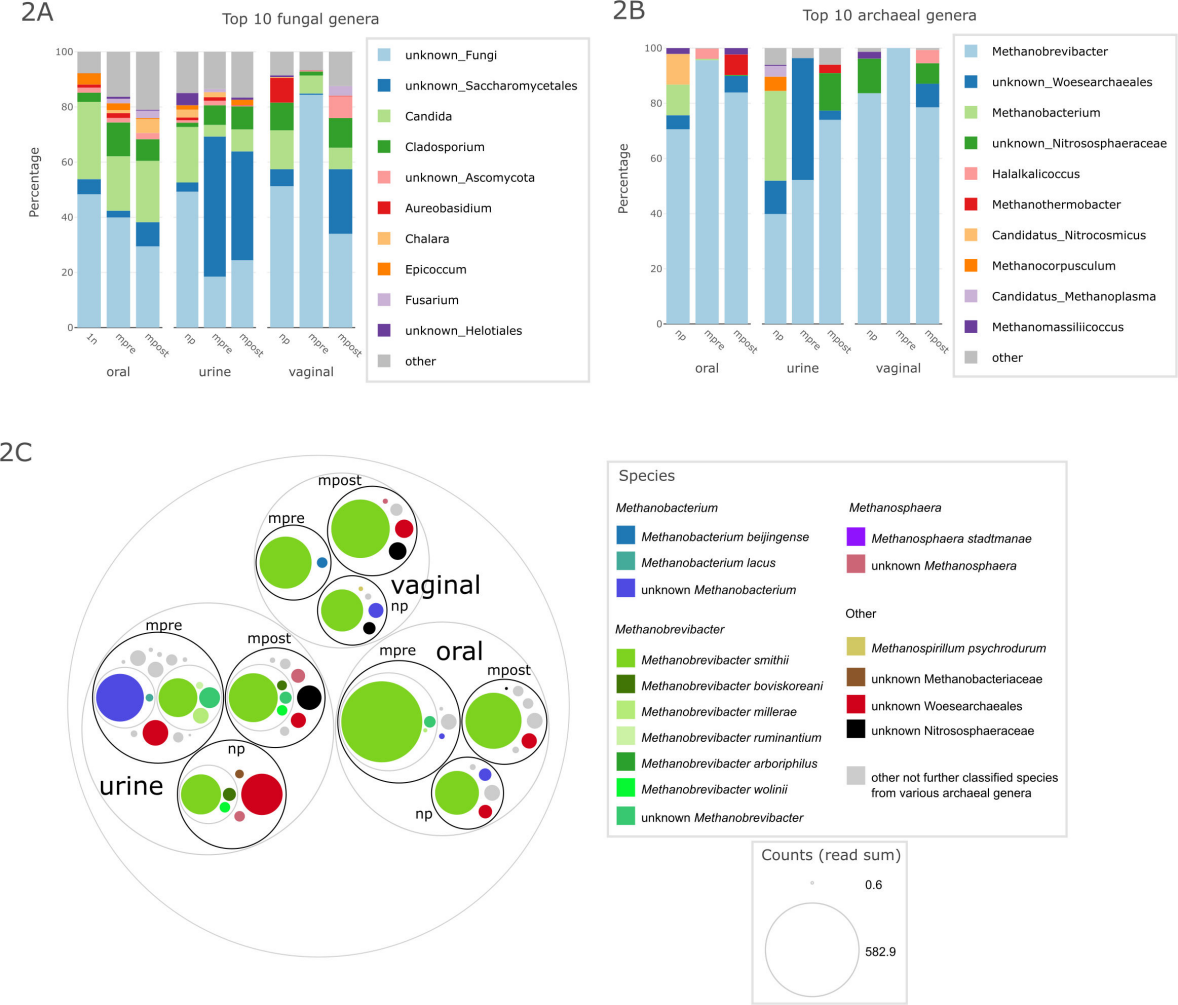
Relative abundance of top 10 (A) fungal genera and (B) archaeal genera, depicted by relative abundance, depicted by sample type and group. (**C**) Circle packing plot of archaeal species depicted by body site and group; the size of the colored dots represents counts of normalized read sums.

Overall, archaeal richness was very low in all samples from all body sites (max. four genera/six species/26 ASVs per individuum and body site; Fig. S7). A total of 25 archaeal genera, 32 species; and 352 ASVs were detected in the data set. *Methanobrevibacter* was the most prevalent archaeal genus in samples from all body sites (Fig. 2B), followed by *Methanobacterium*, which was not found in the mpost group but in all sample types, but especially in urine (Fig. 2C).

The three most abundant ASVs (ASVs 1, 8, and 9) were all classified as *Methanobrevibacter smithii*, which was also the most abundant *Methanobrevibacter* species. These three ASVs accounted for 58.52% of all archaeal reads, 80.24% of *Methanobrevibacter* reads, and 85.52% of all *M. smithii* reads (Fig. 2C). It should be noted that due to insufficient databases for the classification of human-associated archaea, species classification is likely not comprehensive and therefore species information should be taken with caution (20). Classification of *Methanobrevibacter* in particular was further complicated by the fact that the two most common species, *M. smithii* and *Cand.* Methanobrevibacter intestini, cannot be distinguished using V4 16S rRNA gene amplicons (21).

The urinary archaeome did not significantly differ between the groups (Unweighted UniFrac, PERMANOVA, *P* = 0.122). We observed a decrease in Shannon diversity and richness of archaea from np to mpre and mpost (Fig. S7). Whereas the archaeal pattern in np women is more diverse and mainly *Methanobrevibacter* and *Methanobacterium* can be observed, mpre women mainly carry *Methanobrevibacter* and unknown Woesearchaeales (Fig. S8).

The vaginal archaeome carried a variety of archaeal signatures but overall had the lowest Shannon diversity and richness of archaeal genera, species, and ASVs (Fig. S7). Mpre samples contained exclusively *Methanobrevibacter* (*smithii*, ASVs 1, 8, and 9), in contrast to np and mpost samples, which contained other archaeal signatures such as unclassified Nitrososphaeraceae and Woesearchaeales, *Halalkalicoccus*, *Methanomassiliicoccus*, *Methanosphaera,* and *Methanospirillum* (Fig. S9). Interestingly, only five mpre women exhibited any archaea in their vaginal microbiome, and when they did, they were exclusively *Methanobrevibacter* (*smithii*) (Fig. S10). Although the presence of archaea in the vaginal microbiome increased after delivery, no statistically significant differences in the PCoA were observed between the groups (Fig. S5).

In contrast, the oral samples revealed a significant increase in the number (richness) and Shannon Diversity of archaeal ASVs in mpre compared with np and mpost (Fig. S7). This was observed not only in the archaea-specific amplicon approach indicating relative abundance but also in the “universal” approach for which differential abundance could be calculated. *M. smithii* was found significantly increased in mpre compared with mpost (Aldex2, *P* = 0.005, *q* = 0.321) and to np (Aldex2, *P* = 0.003, *q* = 0.300) (Fig. S4). It should be mentioned that, surprisingly, the obtained sequences indeed were classified as *M. smithii* and not as *Methanobrevibacter oralis* although classifications should be taken with caution due to incomplete databases (see above).

In fact, not a single np woman carried *M. smithii* in her oral cavity but 16 mpre women did, whereas after delivery, *M. smithii* was detected in only one mpost woman (Fig. S11). We therefore suggest that the oral niche of a pregnant woman favors the growth of the anaerobic methane-producing archaea *Methanobrevibacter* and *M. smithii* in particular.

In summary, we observed niche restriction for methanogenic archaea in the vagina in pregnancy, whereas the niche in the oral cavity was opened. This indicates that *Methanobrevibacter* species may reflect changes in the ecosystem.

### The oral bacteriome and mycobiome undergo temporary changes with pregnancy

In the oral samples, alpha diversity of the bacteriome did not change substantially with pregnancy status (Fig. 1C). However, beta diversity significantly differed between the groups, as shown by PCoA plots (Unweighted UniFrac, PERMANOVA, *P* = 0.001; Fig. 1A; Fig. S12). It is noteworthy that the mpre samples clustered visibly apart from the other sample groups. We hypothesize that the differences between the groups are mainly due to numerous, highly divergent *Streptococcus* ASVs. *Streptococcus* was identified as the most abundant genus in all three groups, accounting for 43.08% of relative abundance (Fig. S3).

By performing differential abundance analysis, we did not identify a single genus, species, or ASV [with the exception for archaeal *Methanobrevibacter* (*smithii*), see above] that was differentially abundant between mpost and np (with or without BH correction; Fig. S4), indicating that a similar microbial status to prepregnancy was achieved relatively quickly after pregnancy (see Fig. 1C). However, the mpre samples showed a variety of differences (Fig. S4). However, none of these differences remained significant after BH correction, so only trends can be reported (Aldex2, *P* < 0.05, *q* > 0.05). As mentioned above, *Methanobrevibacter* signatures, particularly those of *M. smithii*, were specifically increased in mpre oral samples compared with mpost (*P* = 0.0047, *q* = 0.321) or np (*P* = 0.003, *q* = 0.300) samples. This trend was opposite to that observed for *Streptococcus*. Overall, 12 streptococcal ASVs were increased in the np group and 6 in the mpost group compared with the mpre samples (*P* < 0.050; Fig. S4). Furthermore, the relative abundance of *Streptococcus* ASVs in mpre was rather individual and less uniform than that in np and mpost, as shown by the wider ranges of the confidence intervals (Fig. S4).

It has been reported that the incidence of periodontal disease increases during pregnancy (22), possibly leading to adverse pregnancy outcomes such as preterm birth (23). In our mpre group, we did not observe a significant increase in the relative abundance of the so-called orange and red complex bacteria, which have been shown to account for the initiation and progression of periodontal disease (24) (Fig. S13 S14). It shall be mentioned though that metadata on participants’ dental status was not available, not allowing further interpretation. However, *Methanobrevibacter* load or presence has been previously associated with severe periodontitis (25, 26).

Fungal diversity in the oral cavity increased from np to mpre (Fig. S15). Highly statistically significant differences (Fig. S16, Unweighted UniFrac, PERMANOVA, *P* = 0.001) between the three groups were observed by Adonis. These strong differences can be explained solely by the highly significant differences in two fungal ASVs (ASVs 8 and 9) that were not further classified. ASV8 was highly significantly increased in np compared with mpre (Aldex2, *P* < 0.001, *q* < 0.001) and with mpost (Aldex2, *P* < 0.001, *q* < 0.001), whereas ASV9 was significantly more abundant in mpre than in np (Aldex2, *P* = 0.030, *q* = 0.394). Overall, approximately 50% of all oral fungal ASVs could not be classified. The main members of the oral mycobiome were *Candida albicans* and *Cladosporium cladosporioides* followed by not further classified Saccharomycetales, which were shared between the three groups (Fig. S6).

Overall, we observed temporary fluctuation of the oral microbiome with pregnancy and rapid stabilization of the oral bacteriome and archaeome thereafter.

### The urogenital mycobiome and bacteriome undergo vast changes with and after pregnancy

Due to the local proximity of the urinary and vaginal tracts, an overlap can be biologically expected. We collected noncathetered urine but still specifically asked the participants to collect midstream urine to reduce the contamination risk during sampling. Nevertheless, the overlap was substantial: 77 genera in np, 47 in mpre, and 125 in mpost were shared (Table 2).

**TABLE 2:** Shared and unique bacterial genera between vaginal and urine plotted per group

	Urine unique	Shared urine and vaginal	Vaginal unique	Total
np	470	77	18	565
mpre	302	47	9	358
mpost	174	125	56	355

**Fig 3:**
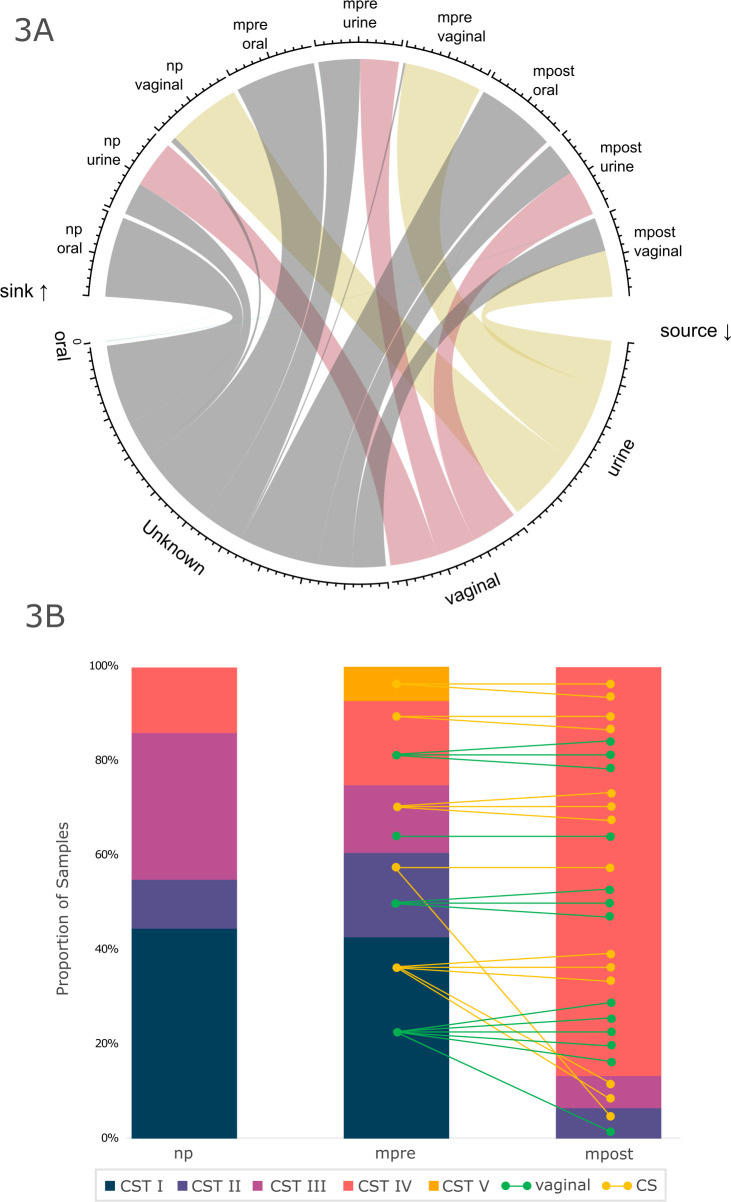
(A) Source tracking; comparison of oral, vaginal, and urine samples with respect to their contribution from oral, urine, vaginal, and unknown sources. (**B**) CST, split per group; lines indicate individuals; colors indicate delivery type; yellow, Cesarean section (CS); green, vaginal.

To numerically assess the microbial exchange between the two sites in the context of all other body sites sampled, we performed source tracking analysis (Fig. 3A; Fig. S17). In all samples, the contribution of the oral microbiome toward the urogenital tract was marginal and did not increase with pregnancy. Overall, the exchange of vaginal and urinary microbial patterns was very clear (Fig. 3A). Notably, the urinary microbiome in mpost samples contributed significantly less to the vaginal microbiome than that in mpre samples [Wilcoxon rank, mpre: 95.25% (median); mpost: 37.18% (median), *P* = 0.002; Fig. S17]. Similarly, we observed a trend toward an overall lower contribution of vaginal samples to urine in samples from mpost (*t*-test, np-mpost, *P* = 0.078; median np: 55.38%, mpre: 38.80%, and mpost: 41.54%).

Furthermore, vaginal and urine (urogenital) samples were analyzed comparatively with respect to their mycobiome and overall microbiome.

For both the vaginal and the urinary mycobiome, the groups clustered statistically significantly on the ASV level (Unweighted UniFrac, PERMANOVA, *P* = 0.001; Fig. S16). The vaginal mycobiome, especially in mpre samples, was predominantly made up of reads that could not be fully classified (Fig. S6 and S18). This was accompanied by a significant decrease in alpha diversity and richness in mpre (mpre-mpost; Shannon *P* = 0.010, richness *P* = 0.035) (Fig. S15) and led to a significant clustering of the groups (Kruskal-Wallis test, adjusted with Bonferroni, *q* < 0.001; Fig. S16). Fungal composition was quite different between groups but only in relative abundance (Fig. S6) and not in differential abundance testing with Aldex2 (Fig. S18). Similar to the vaginal mycobiome, the urinary mycobiome was predominantly unclassified fungal taxa but specifically not further classified Saccharomycetales and Candida (Fig. S6) as already described before (27). Not further classified ASVs were significantly more abundant in np than in mpre and mpost (Aldex2, np-mpre, *q* = 0.044; np-mpost, *q* = 0.161) (Fig. S18). Diversity and especially richness decreased sharply from np to mpre (Shannon, *P* = 0.035; richness, *P* < 0.001) (Fig. S15).

Significant changes in alpha diversity were also observed in bacteria (Fig. 1C, ASV level). Urogenital samples were characterized by a significant increase in Shannon diversity from mpre to mpost. Urine samples showed a continuous increase in alpha diversity from np to mpre to mpost. We observed that in some urine samples, bacterial richness was remarkably high in the np group, with some individuals carrying up to ~150 different bacterial genera. In contrast, vaginal samples had low overall richness (max. 55 different bacterial genera) and diversity. The lowest diversity was observed in vaginal mpre samples, before reaching a higher alpha diversity after birth than in the nonpregnant state (Fig. 1C).

### Delivery has a substantial impact on the urogenital bacteriome driven by a massive loss of *Lactobacillus*

As indicated above, in particular, the urogenital microbiome of postpartum women differed significantly from that of mpre and nonpregnant women, in terms of alpha and beta diversity as well as microbiome composition (Fig. 1A and C). Therefore, beyond composition and diversity, we performed more detailed analyses on these urogenital microbiomes including analysis of CSTs and urine metabolomics.

The vaginal microbiome in postpartum women was very different to that of np or mpre women (Fig. 1A, Unweighted UniFrac; PERMANOVA, *P* = 0.001). Postpartum, alpha diversity strongly increased and was characterized by a massive loss of *Lactobacillus* (*q* < 0.001; Fig. S4; Fig. 1C). This affected not only the three main *Lactobacillus* species representatives (*Lactobacillus crispatus*, *Lactobacillus iners*, and *Lactobacillus gasseri*) but also all other detected species (*Lactobacillus jensenii*, *Lactobacillus delbrueckii* subsp. *delbrueckii*, *Lactobacillus amylovorus*, *Lactobacillus coleohominis*, *Lactobacillus mucosae*, *Lactobacillus ruminis*, and *Lactobacillus sanfranciscensis*). This loss of *Lactobacillus* in the postpartum vaginal microbiome has also been described elsewhere (3) and allowed colonization of the vaginal body site with new appearing bacteria. Accordingly, postpartum vaginal samples had increased abundance of anaerobic bacteria taxa such as *Prevotella*, *Atopobium*, *Streptococcus*, *Anaerococcus*, *Finegoldia*, and *Peptoniphilus*, most of which are also described as prominent members of CST IV (28) or bacterial vaginosis (BV) (29); see Table 3. Women with a CST IV vaginal microbiome may be at a higher risk for BV or other vaginal infections (30, 31).

**TABLE 3:** Increase or decrease of specific bacterial taxa in the vaginal microbiome in CST IV [from reference (3), compared with non-CST-IV], BV [from reference (29), compared with non-BV], and the data set^*a*^

Organism	CST IV	BV	Data set, np to mpost
*Lactobacillus*	Decrease	Decrease	Decrease, ***P < 0.001, ***q < 0.001
*Prevotella*	Increase	Increase	Increase, ***P < 0.001, ***q < 0.001
*Dialister*	Increase	x	Increase, **P = 0.0011, *q = 0.022
*Peptoniphilus*	Increase	x	Increase, ***P < 0.001, **q = 0.0018
*Atopobium*	Increase	Increase	Increase, **P = 0.0066, *q* = 0.077
*Finegoldia*	Increase	x	Increase, *P = 0.0075, *q* = 0.087
*Gardnerella*	x	Increase	Increase, *P* = 0.648, *q* = 0.844
*Mobiluncus*	x	Increase	Increase, *P* = 0.393, *q* = 0.650
*Bifidobacterium*	x	Increase	Increase, *P* = 0.348, *q* = 0.609
*Sneathia*	x	Increase	Not present in the data set
*Streptococcus*	Increase	x	Increase, *P* = 0.014, *q* = 0.131

^*a*^ *P* values and *q*-values are given for Aldex2 comparing np with mpost samples. x means that this genus is not indicated in the regarding literature.

To further analyze this interesting prepartum to postpartum shift in the vaginal microbiome, we classified the vaginal microbiomes into CSTs (Fig. 3B). The predominant CST in mpre and np was indeed CST I, predominated by *Lactobacillus crispatus* (45% in np, 43% in mpre). In 70% of the pregnant cohort, the vaginal microbiome shifted from *Lactobacillus-*predominant CSTs prepartum to CST IV postpartum. Postpartum, CST IV was predominant (23 women, 85%), whereas only five women already had CST IV prepartum. Most women with a prepartum CST other than CST IV switched to CST IV after delivery. This postpartum switch to CST IV has been described previously (3, 14). In the nonpregnant control group, only 14% of the participants had CST IV at the time of sample collection.

Of note, delivery mode (CS versus vaginal delivery) and previous deliveries had no effect on the transition of CSTs from mpre to mpost (χ^2^ test, *P* > 0.506). Similarly, the delivery mode had no effect on the overall composition of the urinary microbiome postpartum (PERMANOVA; vaginal: *P* = 0.188, urinary: *P* = 0.214).

### Urinary metabolic profiles mirror the transition from pre- to postpartum

Urine analysis is an important diagnostic tool in clinics, not only during pregnancy, as changes in the chemical composition of urine can indicate health problems. We subjected mpre and mpost urine samples to metabolomics analysis to assess metabolic situations and correlate with microbial features. Metabolic profiles were generated using untargeted and targeted NMR-based metabolomics.

**Fig 4:**
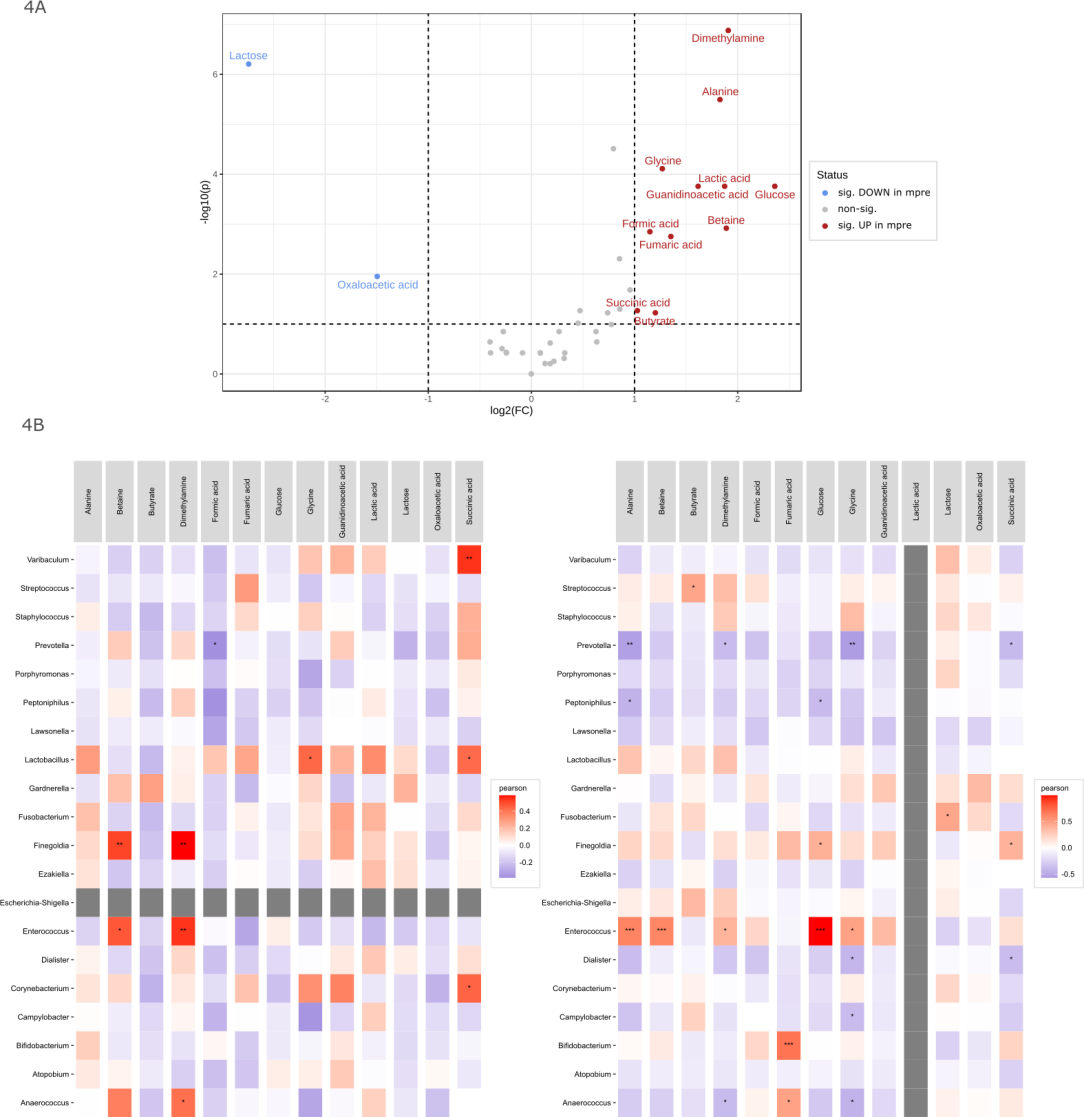
(A) Volcano plot of significantly differentially abundant metabolites between mpre and mpost. (B) Pearson correlation of top 20 abundant bacterial genera with 13 metabolites in urine samples which are differentially abundant between mpre and mpost. Left, mpre; right, mpost. The gray entries for Escherichia-Shigella in mpre and lactic acid in mpost reflect the absence of those features or metabolites in the subset. Significance levels are indicated with asterisks for ***P  <  0.001, **P  <  0.01, and *P  <  0.05.

When comparing mpre and mpost samples, a significantly higher lactose content was detected in mpost (*q* < 0.001, *t*-test after log transformation, paired samples; Fig. 4A), indicating active lactation. Postpartum women who did not breastfeed (*n* = 2) lacked detectable lactose in their urine. In addition, a significant increase in oxaloacetic acid was observed (Fig. 4A, *q* = 0.011), which might be explained by an altered metabolic status during breastfeeding (32). These two compounds were also significantly higher in postpartum samples compared with nonpregnant controls (lactose: *q* < 0.001; oxaloacetic acid: *q* < 0.001; *t*-test after log transformation, unpaired samples), but no other compounds were found to be significantly different. However, the urine samples from mpre women had significantly increased levels of dimethylamine, alanine, glycine, lactic acid, and other compounds, as shown in Fig. 4A.

Prepartum, none of the individuals carried the genera *Escherichia-Shigella. Finegoldia*, *Enterococcus*, and *Anaerococcus* were correlated with betaine and dimethylamine (Fig. 4B, i). In addition, we found significant correlations for *Varibaculum* with succinic acid (*q* = 0.003), *Lactobacillus* with glycine and succinic acid, and *Corynebacterium* with succinic acid. Postpartum (Fig. 4B, ii), *Enterococcus* correlated strongly with alanine (*q* < 0.001), betaine (*q* < 0.001), and glucose (*q* < 0.001) as well as with dimethylamine (*q* = 0.047) and glycine (*q* = 0.012). Lactic acid was not detectable in any of the mpost urine samples. Fumaric acid was positively correlated with *Bifidobacterium* (*q* < 0.001) and *Anaerococcus* (*q* = 0.012). *Prevotella*, *Porphyromonas*, *Peptoniphilus*, and *Lawsonella* were weakly negatively correlated with all 13 metabolites in both mpre and mpost.

In healthy situations, the production of lactic acid lowers the pH, which prevents the growth of other microbes and promotes the acid-tolerant lactobacilli. Indeed, lactic acid was reduced in mpost samples (Fig. 4Aand B, ii), highlighting the loss of acid-producing *Lactobacillus* activity postpartum.

Although the increase of lactose and oxaloacetic acid in urine might be correlated with one of the 20 most abundant microbial genera, lactose in combination with the acid might favor the growth of lactobacilli in the long term.

## DISCUSSION

In this paper, we demonstrated the dynamic transition of the maternal microbiome and urinary metabolome from the nonpregnant to the perinatal period. We revealed abrupt changes in the bacterial, fungal, and archaeal components of the microbiome in the oral and urogenital (urine and vaginal) body regions of 30 women from the pregnant state to 1 month postpartum and placed them in context to the microbiome of nonpregnant women. Understanding the normal postpartum microbiome transition and the influence of maternal (modifiable) factors could help develop prevention strategies for women who tend to develop typical microbiome-associated postpartum health problems or infections.

Pursuing a molecular approach that allowed us to capture nonbacterial components of the microbiome added interesting aspects to the microbiome transition. The overall archaeome profiles found in the urogenital tract (mainly Methanobacteriota and Halobacteriota) were consistent with the literature (33) but were not indicative of pregnancy status. Other signals reflected the pregnancy status well: we detected *Methanobacterium* almost exclusively in nonpregnant women (all four body sites examined). Even clearer evidence was provided by the major player among the archaea, *Methanobrevibacter*. The abundance of this archaeon was significantly increased in the oral cavity of pregnant women but rapidly decreased to prepregnancy levels after delivery. Indeed, alpha diversity of the oral microbiome increases with pregnancy, and an increase of pathogenic taxa has also been reported (34). Methanogenic archaea are known to effectively support fermenting microorganisms by eliminating inhibiting end products (especially H_2_ and CO_2_) (35). Therefore, increased methanogenic archaea could be a sign of excessive growth of bacterial anaerobes at this stage, which might be associated with periodontitis. Indeed, pregnant women are more prone to dental problems (36).

Interpretation of signals from the mycobiome was difficult due to the large number of taxonomically unclassified reads that were received. This again highlights the lack of appropriate high-throughput methods and mycobiome databases for read annotation, especially from samples outside the gastrointestinal tract. Although the mycobiome analyses in our approach were not specifically targeting the mycobiome (e.g., adjustments in cell lysis, selection of primers, or removal of host DNA could have improved results), we still obtained meaningful information: the overall mycobiome profile changed with pregnancy status, consistent with observations from previous, gut-focused studies (37). This change was also reflected by unknown Saccharomycetales that increased in urine samples with pregnancy.

Indeed, pregnant and postpartum women are at higher risk for health problems at these body sites, such as periodontitis (8, 9), bacterial vaginosis (29), or fungal infection of the vagina (13, 30, 31). Any aspects that could contribute to a rapid return to a microbiome composition typical of a healthy, nonpregnant state could help reduce these microbiome-related complications, which could also affect maternal recovery.

In agreement with other reports (3, 14, 38, 39), we clearly demonstrated that the vaginal microbiome does not return to the nonpregnant state 1 month after delivery but that remains substantially altered, mainly due to the decrease of *Lactobacillus*. This decline has already been reported (14), and it can be explained by a decrease in estrogen in the female body due to impaired ovarian activity after delivery and during lactation (3). Decreased abundance of lactobacilli, especially in the first 6 weeks postpartum, may also be due to the lochia, which has previously been characterized as alkaline and therefore may restrict the growth of *Lactobacillus* species (3). Loss of lactobacilli is also accompanied by an increase in other, often opportunistic pathogenic bacteria, leading to the predominance of CST IV in postpartum women, which is generally associated with urogenital infections (40, 41). During the puerperal time, women are at higher risk of vaginal postnatal infections due to the large wound area, as well as other infections such as endometritis when vaginal opportunistic bacteria enter the upper genital tract, or urinary infections (14, 42). Probiotics treatment was already investigated in multiple other studies to prevent or fight vaginal infections (43–45). It needs to be further investigated how and to what extent administration of probiotics postpartum is capable to accelerate recovery of the postpartum vaginal microbiome.

Of note, the mode of delivery did not appear to affect the postpartum vaginal microbiome. Since many women in this cohort who delivered vaginally had birth tears and all women who delivered by cesarean section were treated with antibiotics, we would have expected a separation of their microbiomes, but this effect was not observed.

Source tracking analysis indicated a strong exchange between the vaginal and urinary microbiome, with the overall effect of the urinary microbiome on the vaginal microbiome being greater than *vice versa*. Thus, not only microorganisms but also important metabolic compounds, such as lactose, could be transferred. The greatly increased lactose content in postpartum urine might contribute to the recovery of lactobacilli throughout the urogenital tract. Indeed, lactose added to fecal samples could specifically increase the abundance of lactobacilli in *in vitro* experiments (46).

In addition to lactose, oxaloacetic acid was also increased in postpartum urine. Lactose is an important indicator of active lactation and might also be released from microbial metabolism of human milk oligosaccharides excreted with the urine (13). However, the origin of oxaloacetic acid is less clear. The increased excretion of unused oxaloacetic acid could reflect an increased catabolic state during lactation (32). We hypothesize that the urinary metabolome retains this signature after birth as long as the woman is breastfeeding.

In general, the female body is in an exceptional state after birth and still when the woman is breastfeeding, even months after delivery. As long as lactation hormones are produced, metabolism and physiology are altered compared with a nonpregnant and nonbreastfeeding state. Despite the fact that breastfeeding has numerous beneficial effects not only on the child but also on the mother, it might decelerate the return of the female body and its microbiome to the nonpregnant state (14). This is a very interesting research question, and longitudinal sampling of postpartum women who breastfeed and who do not breastfeed is needed to clarify this hypothesis.

### Limitations

This study is based on a relatively small sample size of 30 participants per group. Still, we are able to depict meaningful aspects of the perinatal microbiome especially by highlighting the understudied fungal and archaeal part of the microbiome in three body sites of these participants.

### Conclusion

In addition to the postpartum shift of *Lactobacillus* population in the vagina, which remains to be explored, additional work should be invested in the use of probiotics to improve postpartum maternal health. To avoid potentially long-term establishment of unhealthy CSTs and associated vaginal/urinary bacterial or fungal infections, we would suggest increasing *Lactobacillus* numbers in the vaginal niche, for example, by administering probiotics to naturally accelerate the establishment of a healthier vaginal microbiome that is better able to fight pathogens. Additionally, it would be important to explore why some women keep an optimal vaginal microbiome state after delivery characterized by a predominance of *Lactobacillus* species, as this could provide insights in the mechanisms that could be targeted for promoting an optimal vaginal microbiome postpartum. Overall, it remains clear that most of the research has focused on the health of the child, whereas the important recovery of the mother has not been fully elucidated, especially with regard to the nonintestinal microbiome.

## MATERIALS AND METHODS

### Study design

Fifty-nine participants were enrolled in this pilot study to explore the microbial changes that occur with pregnancy, by comparing the microbiome of different body sites of a cohort of healthy pregnant women (*n* = 30) with the microbiome of the same body sites of a cohort of healthy age-matched nonpregnant women (*n* = 29). All recruited participants were healthy, were 18 years of age or older, and consent to all aspects of the protocol. Participants were excluded if they had any recent genitourinary infections, if they had taken any antibiotic/probiotic treatment in the last 6 months, if they smoked or took prescription medicines, and if they had HIV or HCV. Pregnant participants were also excluded if they had multiple pregnancy, membrane rupture longer than 12 h, prepregnancy diabetes type 1 or 2, gestational diabetes mellitus, prepregnancy hypertension, or Preeclampsia/HELLP. Metadata from all participants are listed in the GitHub Repository (47). All participants were recruited at the Medical University of Graz, Styria, Austria, from 2017 to 2020.

### Sample collection and processing

For pregnant participants, samples were collected at two time points, 1 to 2 weeks before delivery (prepartum) and 1 month postpartum. The following samples were collected for all three study groups: urine, vaginal swabs, and oral swabs. Samples were collected by the participants after they had been clearly instructed on how to collect and store the samples.

Approximately 20 mL of midstream urine was collected in sterile collection tubes at a not-further-specified time of the day. Vaginal and oral samples were collected using FLOQSwabs (Copan). Oral samples were collected from the cheek buccal mucosa. All samples were stored in the fridge without addition buffer added and were then transported to the lab on ice and stored at −80°C until further processing.

Genomic DNA was extracted from the urine, oral, and vaginal specimens using the QIAamp DNA Mini Kit (QIAGEN) with some modification: before the extraction with the kit, the urine samples were centrifuged at 4,400 × *g* for 15 min and the supernatant was removed except for 500 µL that was used to resuspend the pellet. Five hundred microliters of Lysis Buffer (sterile filtered, 20 mM Tris-HCl pH 8, 2 mM Na-EDTA, 1,2% Triton X-100) was added to the vaginal and oral swabs. To all the samples, 50 µL of lysozyme (10 mg/mL) and 6 µL of mutanolysin (25 KU/mL) were added, followed by an incubation at 37°C for 1 h. The obtained mix was transferred to Lysing Matrix E tubes (MP Biomedicals) followed by a step of mechanical lysis at 5,500 rpm for 30 s two times using the MagNA Lyser Instrument (Roche). After the mechanical lysis, the samples were centrifuged to separate the beads from the supernatant at 10,000 × *g* for 2 min. Afterward, the DNA was extracted according to the provided instructions. The DNA was eluted in 100 µL of Elution Buffer for the vaginal and in 60 µL for the urine and oral samples. For all samples, the genomic DNA concentration was measured using Qubit HS (Thermo Fischer Scientific). Most urine samples had a DNA concentration under the detection limit (<0.1 ng).

Additional swabs and tubes filled with buffer served as negative controls. Negative controls were processed alongside during DNA extraction, PCR, sequencing, and quality control.

Statistical analyses on the metadata of the cohort were performed in SPSS using either *t*-test, Mann-Whitney U test (independent samples), or Fisher’s exact *t*-test as indicated in Table 1.

### PCR amplification

The obtained genomic DNA was used to amplify the V4 region of the 16S rRNA gene using Illumina-tagged primers, 515FB and 806RB (Table 4). Those primers target and partially cover the 16S rRNA gene of both bacteria and archaea, but mainly the bacterial part, and are therefore named “universal” or bacterial. In order to specifically identify the archaeal communities present in the samples, a nested PCR was performed using the primer combination 344F-1041R/519F-Illu806R as described previously (48). For the fungal communities, the ITS2 region was amplified using the primers ITS86F/ITS4. The PCR reactions were performed in triplicates in a final volume of 25 µL containing TAKARA Ex Taq buffer with MgCl2 (10×; TAKARA Bio Inc.), primers 200 nM, dNTP mix 200 µM, TAKARA Ex Taq Polymerase 0.5 U, water (LiChrosolv; Merck), and DNA template (1–2 µL of genomic DNA). The PCR amplification conditions are listed in Table 5.

**TABLE 4:** Primer pairs used for “universal,” archaeal, and fungal PCRs

Approach and target	Name	Sequence (5′−3′)	Reference
PCR “Universal”	515FB	GTGYCAGCMGCCGCGGTAA	(49)
	806RB	GGACTACNVGGGTWTCTAAT	(49)
PCR Archaea I/II	344F	ACGGGGYGCAGCAGGCGCGA	(50)
	1041R	GGCCATGCACCWCCTCTC	(50)
PCR Archaea II/II	519F	CAGCMGCCGCGGTAA	(50)
	806R	GGACTACVSGGGTATCTAAT	(50)
PCR Fungi	ITS86F	GTGAATCATCGAATCTTTGAA	(51)
	ITS4	TCCTCCGCTTATTGATATGC	(51)

**TABLE 5:** PCR settings for the primer pairs used

Target gene	Primer pair	Initial denaturation	Denaturation	Annealing	Elongation	Final elongation	No. of cycles
“Universal” (16S rRNA gene)	515FB - 806RB	3′, 94°C	45″, 94°C	1′, 50°C	1′ 30″, 72°C	10′, 72°C	35
Archaea (16S rRNA gene)	344F-1041R	5′, 95°C	30″, 94°C	45″, 56°C	1′, 72°C	10′, 72°C	25
	519F-806R	5′, 95°C	40″, 95°C	2′, 63°C	1′,72°C	10′, 72°C	30
Fungi (ITS region)	ITS86F - ITS4	1′, 94°C	30″, 94°C	30″, 56°C	30″, 68°C	10′, 68°C	35

### Amplicon sequencing, bioinformatics, and statistical analysis

Library preparation and sequencing of the amplicons were carried out at the Core Facility Molecular Biology, Center for Medical Research at the Medical University Graz, Austria. In brief, DNA concentrations were normalized using a SequalPrep normalization plate (Invitrogen), and each sample was indexed with a unique barcode sequence (eight cycles index PCR). After pooling of the indexed samples, a gel cut was carried out to purify the products of the index PCR. Sequencing was performed using the Illumina MiSeq device and MS-102-3003 MiSeq Reagent Kit v3-600cycles (2 × 300 cycles). The obtained 16S rRNA gene amplicon data are available in the European Nucleotide Archive under the study accession number PRJEB65415.

The analysis of the 16S rRNA gene amplicon data was performed using QIIME2 (52) 2021.1-12 as described previously (53). Removal of primers and chimeras and quality filtering were performed with the DADA2 algorithm (54) for truncation (-p-trunc-len-f 200 –p-trunc-len-r 150) and denoising to generate ASVs. Taxonomic classification (55) was based on the SILVA 138 database (56) for the “universal” and archaeal approach and the UNITE database (57, 58) for the ITS approach. The obtained feature table and taxonomy file were used for further analysis. Contaminating ASVs were determined and removed by decontam v 1.13 (59) in R (60), running *iscontaminant* in *prevalence* mode with a threshold of 0.5. After this step, positive and negative controls were removed from the data sets. Additionally, ASVs classified as chloroplast and mitochondria were removed.

For normalization, different approaches were used for the three bacterial, fungal, and archaeal data sets based on their composition. Scaling with ranked subsampling normalization was run in QIIME2 (52) with *c*_min_ = 1,500 for the bacterial data set and with *c*_min_ = 100 for the fungal data set. The archaeal data set was normalized with total sum normalization. The number of samples that were analyzed and the number of how many samples were kept after normalization are listed in Fig. S1.

Differentially abundant taxa between the three groups were defined by q2-aldex2 (61–63) in QIIME2 (52) using standard settings, CLR (centered log-ratio) transformed in R (60), and plotted in boxplots in R [packages: ggplot2 (64), dplyr (65), and reshape (66)].

Several analysis steps were performed with Microbiome Explorer in R (67), e.g., for stacked bar plots and boxplots of alpha diversity. Statistics for alpha diversity were performed using the Kruskal-Wallis test of independent samples, adjusted with Bonferroni. PCoA were calculated and plotted in R (60) using the vegan package (68) and an Unweighted UniFrac distance matrix. Differences in beta diversity between groups were assessed with a PERMANOVA on the unweighted UniFrac distances as implemented in the vegan package (68) in R (60) accordingly.

Source tracking was performed with Sourcetracker2 (69) (standard settings) with rarefaction done at 1,500 sequences. Data were then visualized by RAWGraphs (70), based on the median of each sample set.

Sequences classified as the *Lactobacillus* genus were further classified to the species level to allow the clustering of the vaginal microbiome into CSTs. The classification was performed by classification through EzBioCloud (71). The CSTs were classified using the VALENCIA (VAginaL community state typE Nearest CentroId clAssifier) classifier (28) as described in the GitHub tutorial (72). The biom table used to run the classifier VALENCIA as well as the obtained output can be found in the GitHub Repository.

### NMR metabolomics on urine samples

A subset of 87 urine samples from all three groups was analyzed in house with untargeted NMR for several metabolites as described previously (73). In short, methanol water was added to the samples and cells were lysed, lyophilized, and mixed with NMR buffer. NMR was performed on an AVANCETM Neo Bruker Ultrashield 600 MHz spectrometer equipped with a TXI probe head at 310 K and processed as described elsewhere (74). NMR data were analyzed using MetaboAnalyst (75), following the protocol for paired samples (comparison mpre to mpost; log transformation).

Significantly differentially abundant metabolites were correlated with CLR-transformed counts of bacterial genera in R (60) and plotted in heatmaps with ggplot2 (64).

### Visualization and sample overview

The circle packing plot about archaeal occurrence was created with rawgraphs.io (70). All figures were aligned in Inkscape v 1.1 (URL: https://inkscape.org/en/). Codes and all data tables are available in our GitHub Repository https://github.com/CharlotteJNeumann/PerinatalMicrobiomeTRAMIC (47). An overview of the available data is displayed in two figures: Fig. S1 is following the STORM guideline and was created with drawio.com (URL: https://drawio.com). Fig. S2 displays the data available per sample and individuum.

### Reproducibility

We conducted a prospective pilot study whereas sample size was not predetermined beforehand. Randomization and blinding of the investigators were not foreseen in the chosen study setup. A full study flow chart is provided in Fig. S1. Participants 13 and 17 were excluded from the study due to incomplete sampling, and all data from stool were excluded as no stool samples were collected in the mpost group, and therefore, this body site could not be sufficiently examined with the focus of this manuscript. Overall, the study is considered to be only partially reproducible, as the data are dependent on the study cohort, which was only sampled once within this study, and sampling of cohorts at the same time window cannot be repeated. However, starting from the raw sequencing data, the analysis is fully reproducible, and all required data, scripts, and details are provided.

## Data Availability

A STORMS (Strengthening The Organization and Reporting of Microbiome Studies) checklist (76) is available at 10.5281/zenodo.10458418. Data are available in the GitHub Repository https://github.com/CharlotteJNeumann/PerinatalMicrobiomeTRAMIC (47). The obtained 16S rRNA gene amplicon data are available in the European Nucleotide Archive under the study accession number PRJEB65415.

## SUPPLEMENTAL MATERIAL

Supplemental material spectrum.00147-24-s0001.docx Fig. S1 to S18.

ASM does not own the copyrights to Supplemental Material that may be linked to, or accessed through, an article. The authors have granted ASM a non-exclusive, world-wide license to publish the Supplemental Material files. Please contact the corresponding author directly for reuse.
