# Supplementary material for: The dynamics of the female microbiome: unveiling abrupt changes of microbial domains across body sites from prepartum to postpartum phases
Source: Microbiol Spectr. 2024 Jun 25;12(8):e00147-24. doi: 10.1128/spectrum.00147-24 (PMC11302012; doi:10.1128/spectrum.00147-24)
Supplement: Supplemental material — Fig. S1 to S18. [file spectrum.00147-24-s0001.docx]

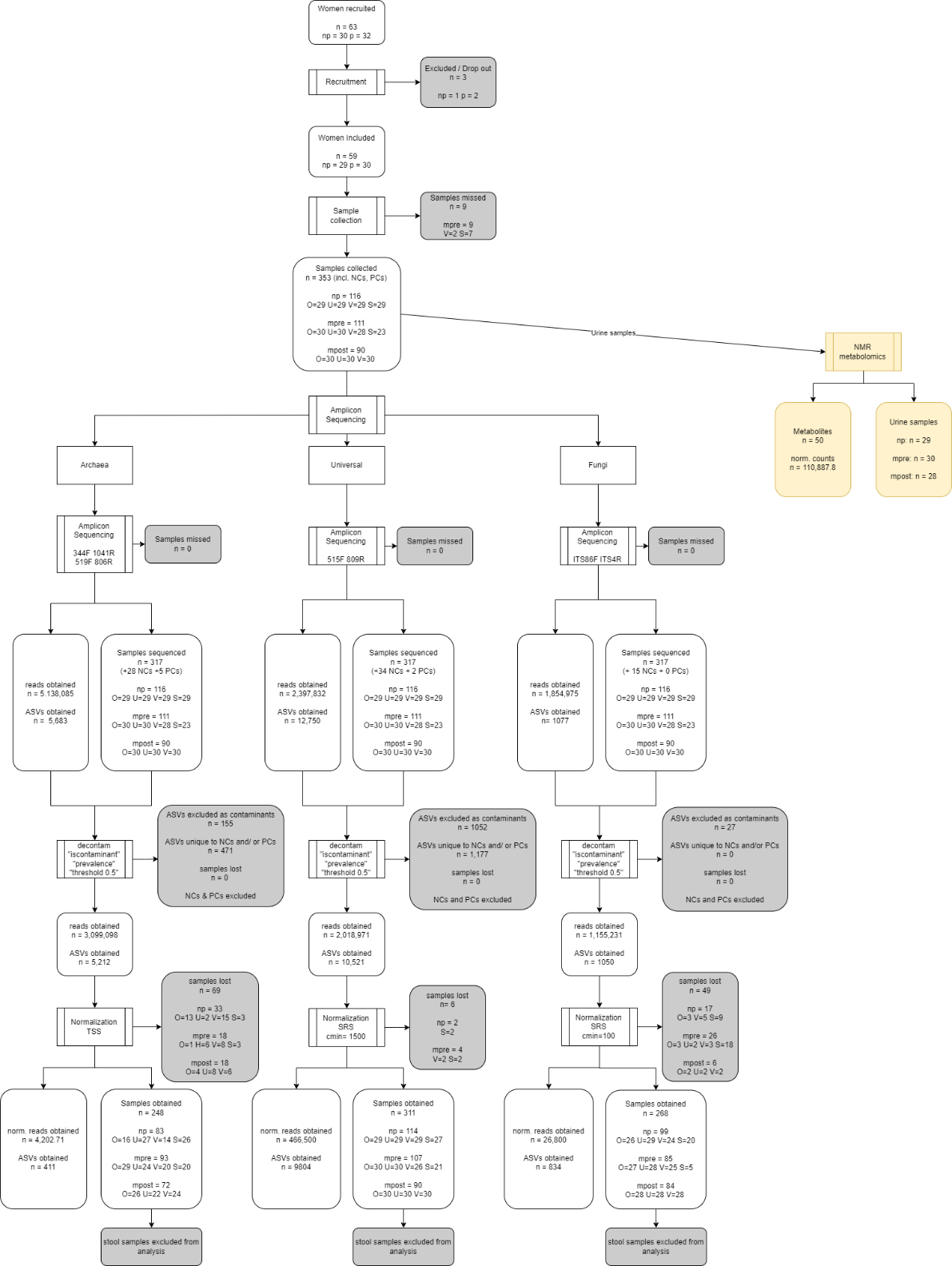
Suppl. Fig. 1: flowchart according to the STORM pipeline, showing sample and read numbers for data analyses steps


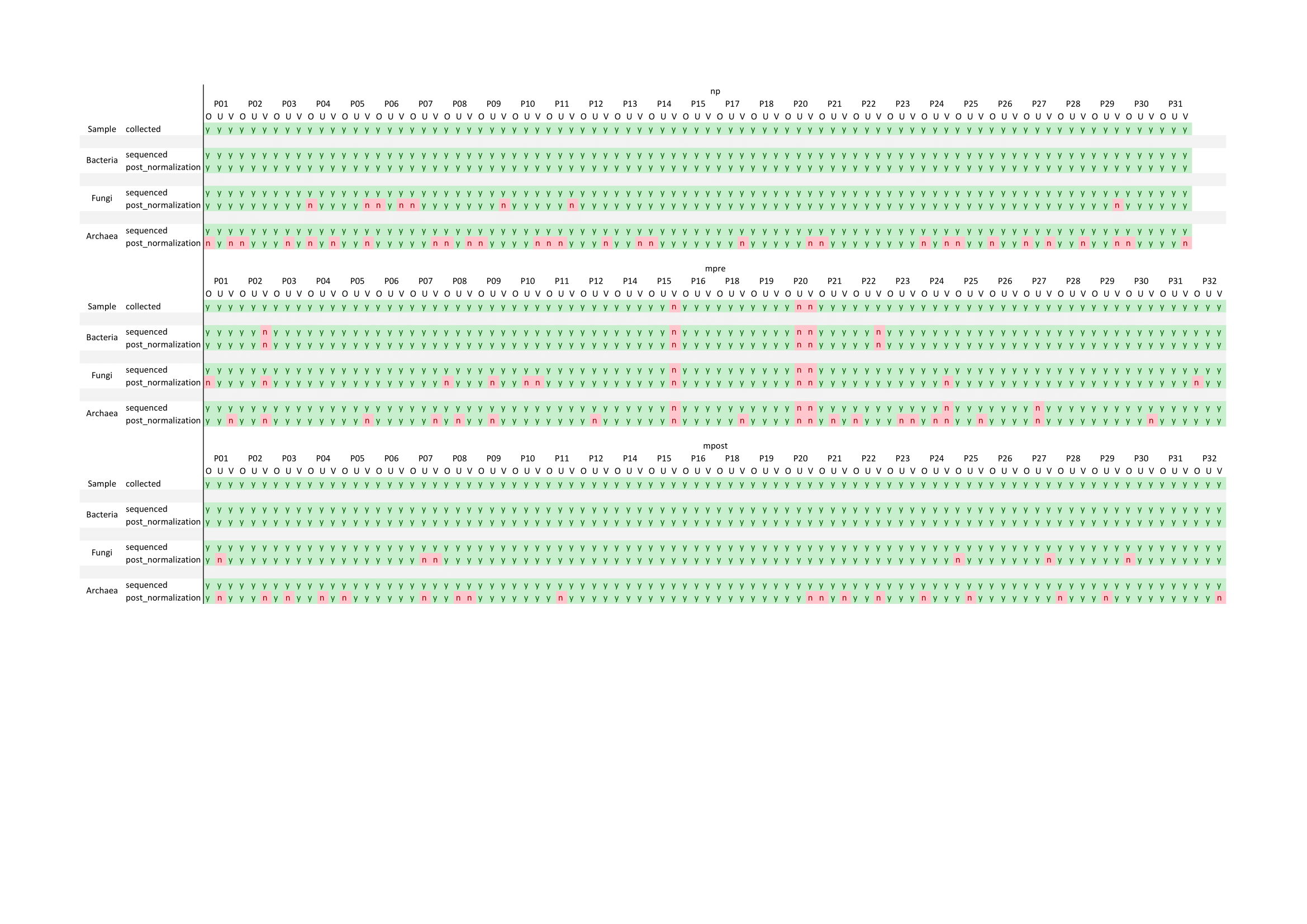


Suppl. Fig. 2: sample overview for nonpregnant (up), mpre (middle) and mpost (down) for single individuals; it is shown if samples are available (y, green) or not (n, red) at the steps of sample collection, sequencing and post-normalization, each for bacteria, fungi and archaea.


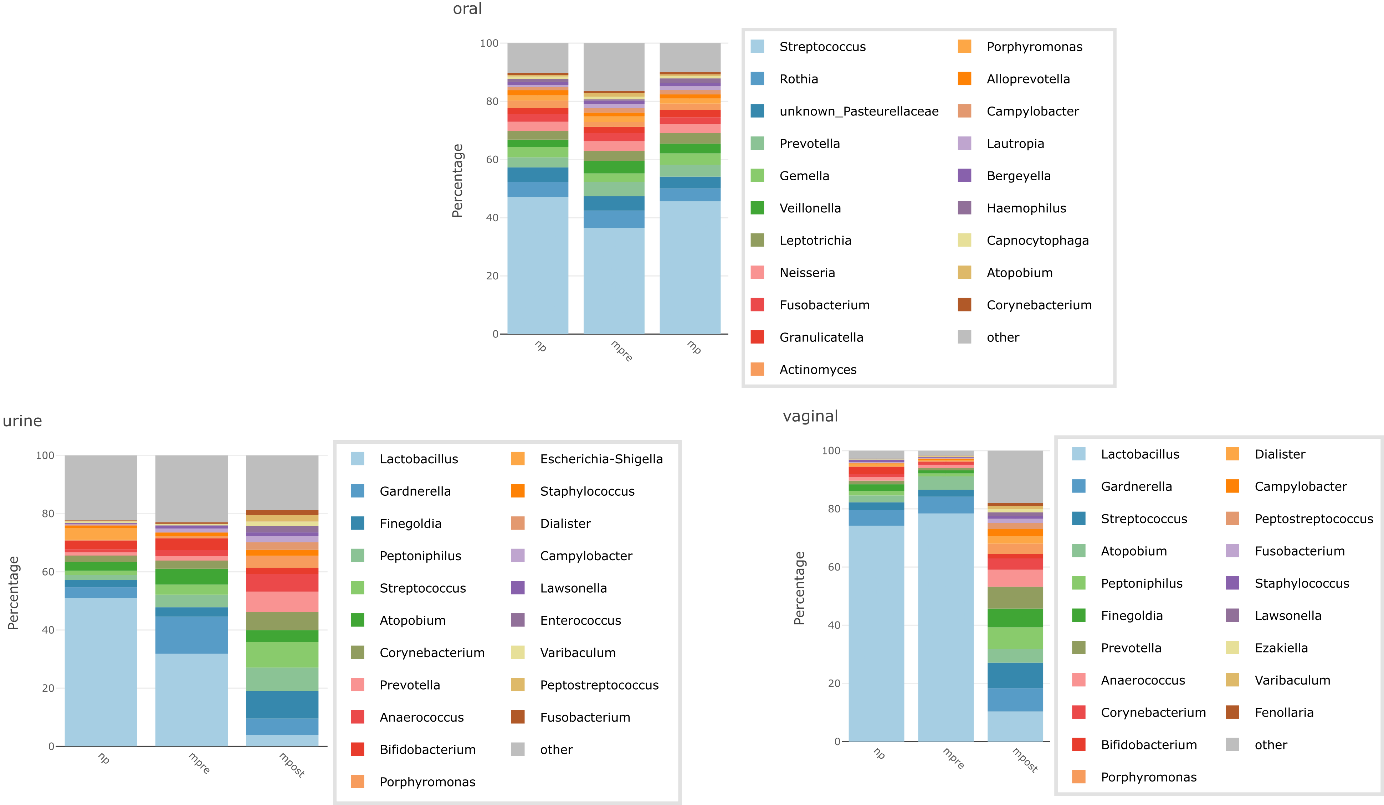


Suppl. Fig. 3: stacked bar plots showing relative abundances of the top 20 most abundant bacterial genera depicted per group for oral, urine and vaginal samples.


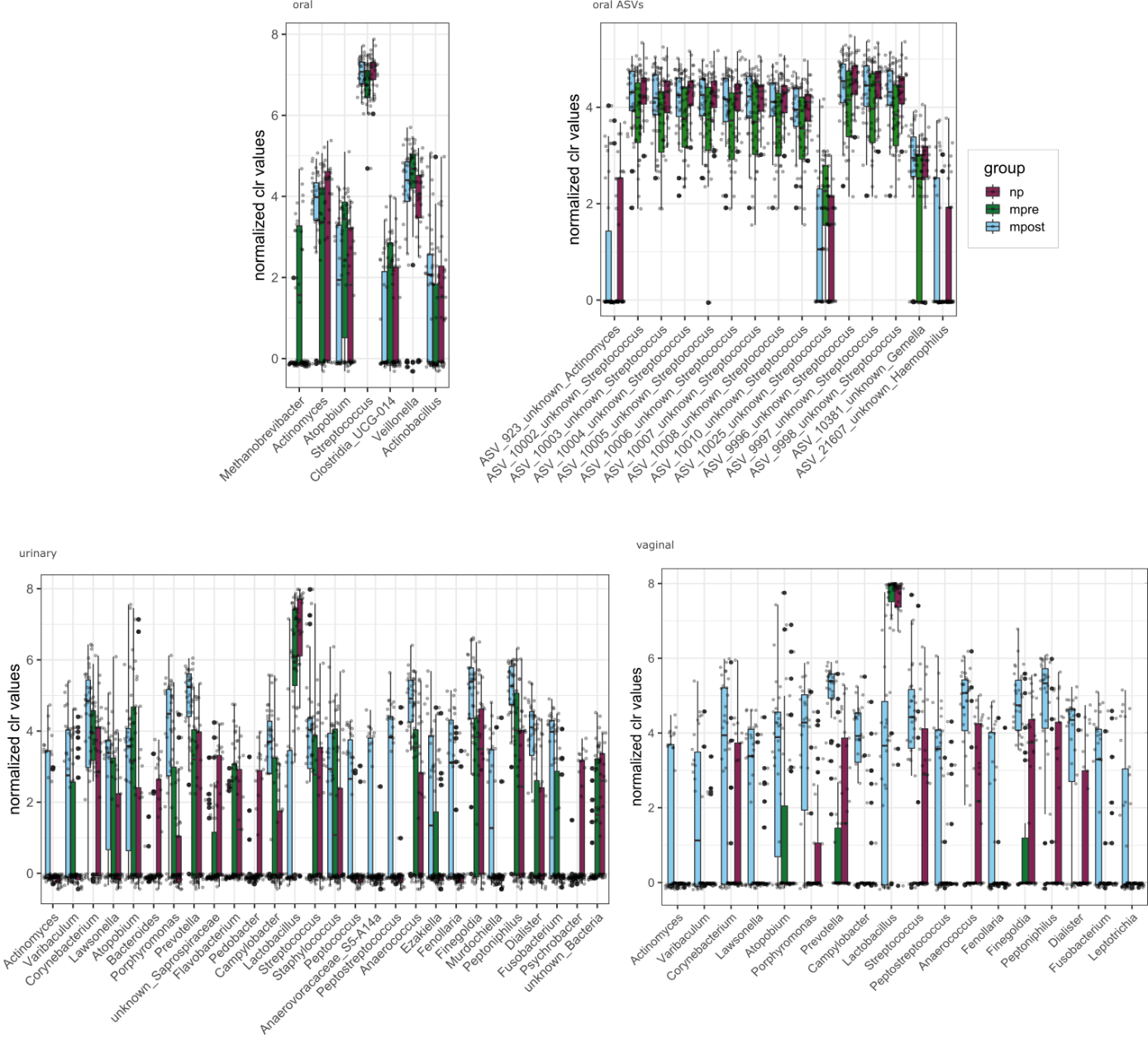


Suppl. Fig. 4: differential abundance boxplots of CLR transformed values on bacterial genera (Aldex2) for oral, urine and vaginal samples. For oral, additionally ASVs are also depicted


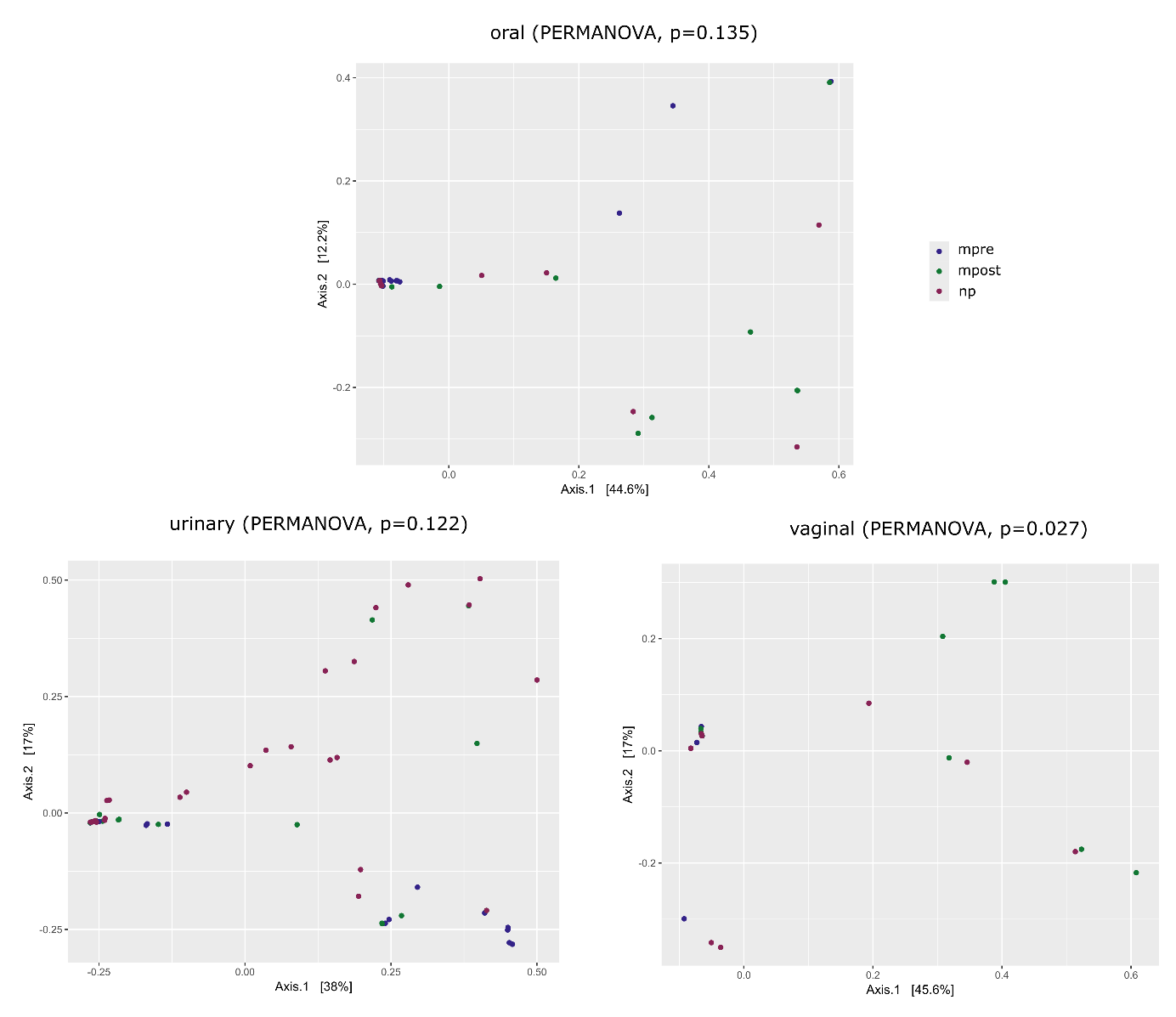


Suppl. Fig. 5: Principal Coordinate Analysis (PCoA) for groups (np, mpre, mpost) for archaeal ASVs with Unweighted UniFrac as distance Matrix and p-values


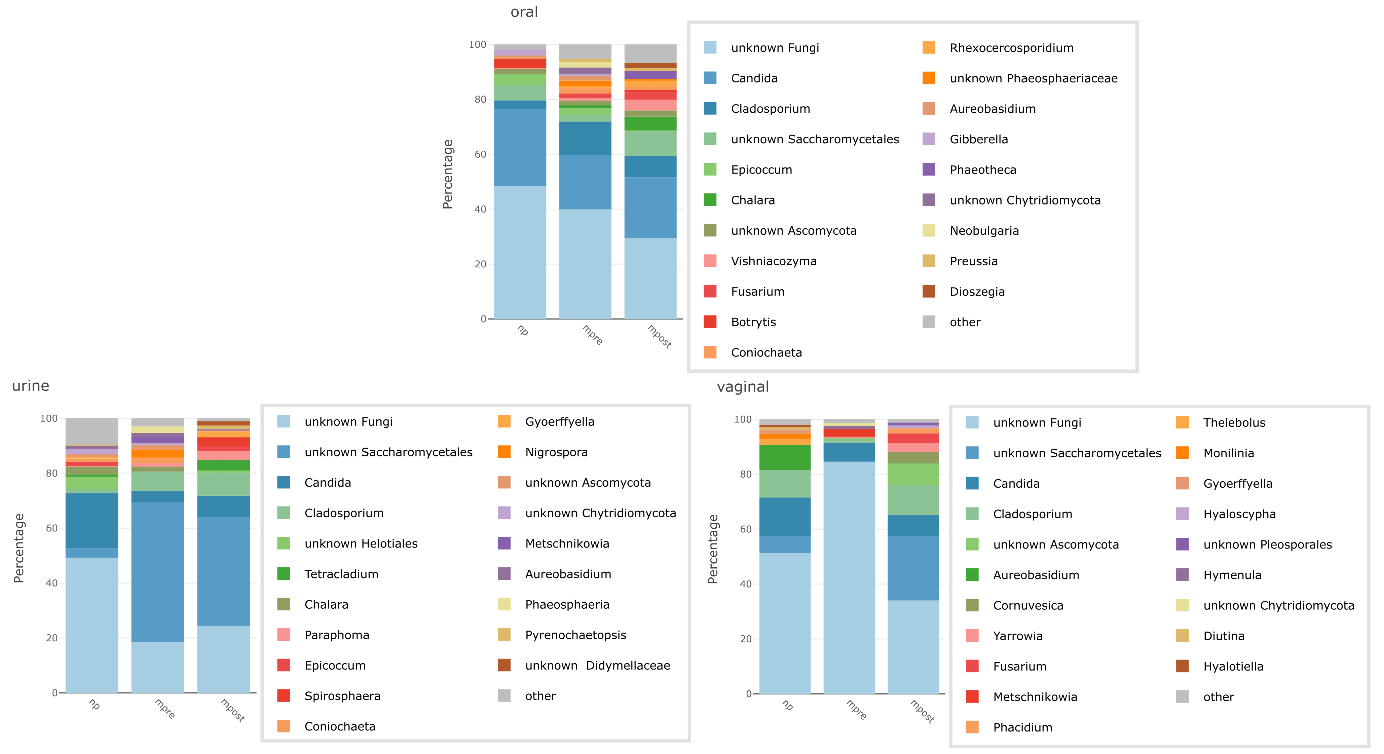


Suppl. Fig. 6: stacked bar plots showing relative abundances of the top 10 or 20 most abundant fungal genera depicted per group for oral, urine and vaginal samples.



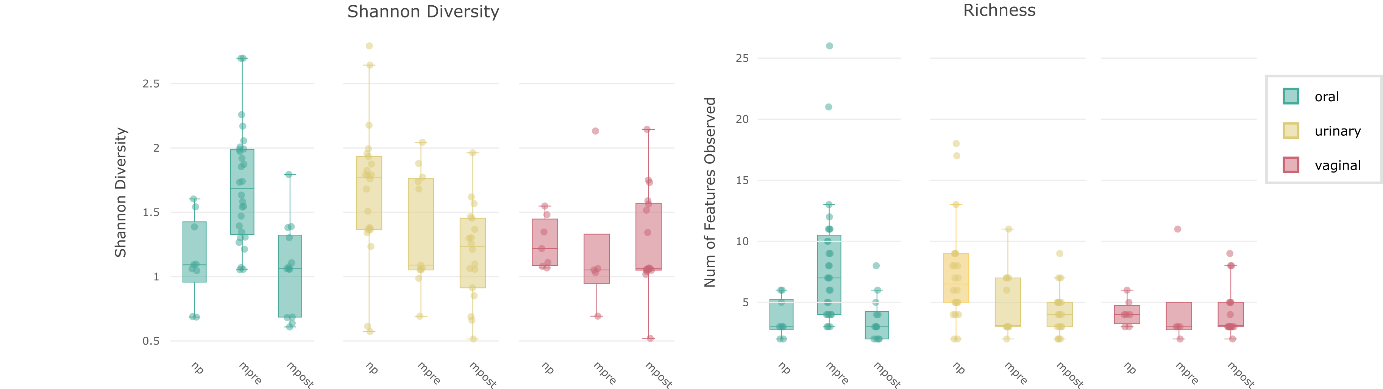
Suppl. Fig. 7: alpha diversity of the archaeal microbiome on ASV level, archaea nested PCR approach: Shannon diversity and richness, depicted per body site and split by group *p* < 0.05*, *p* < 0.005**, *p* < 0.001***


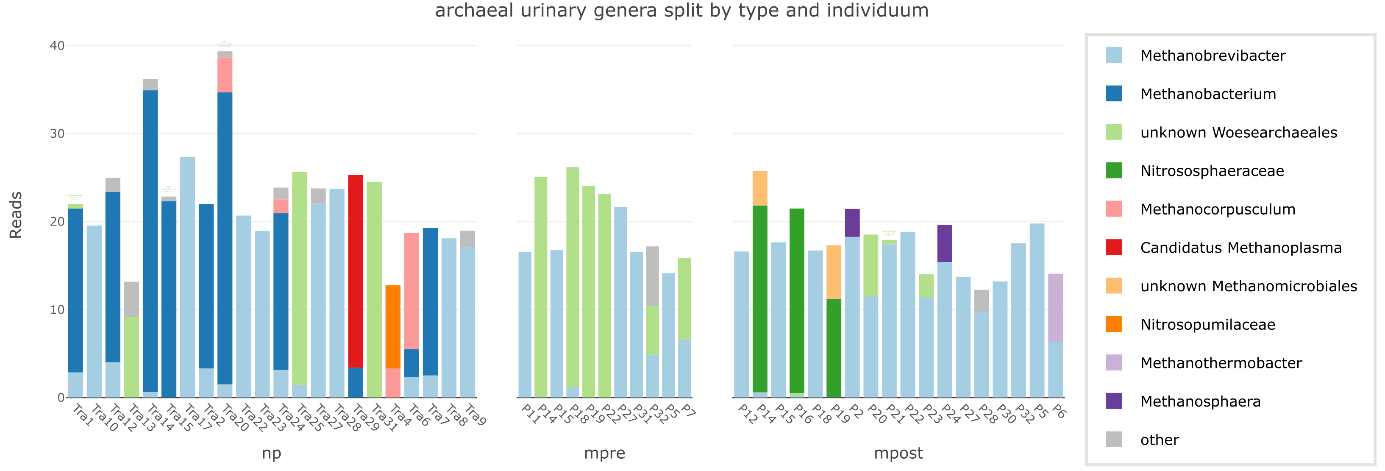


Suppl. Fig. 8: occurrence (reads, archaea nested PCR approach) of archaeal genera in the urinary microbiome, split by group per individuum.


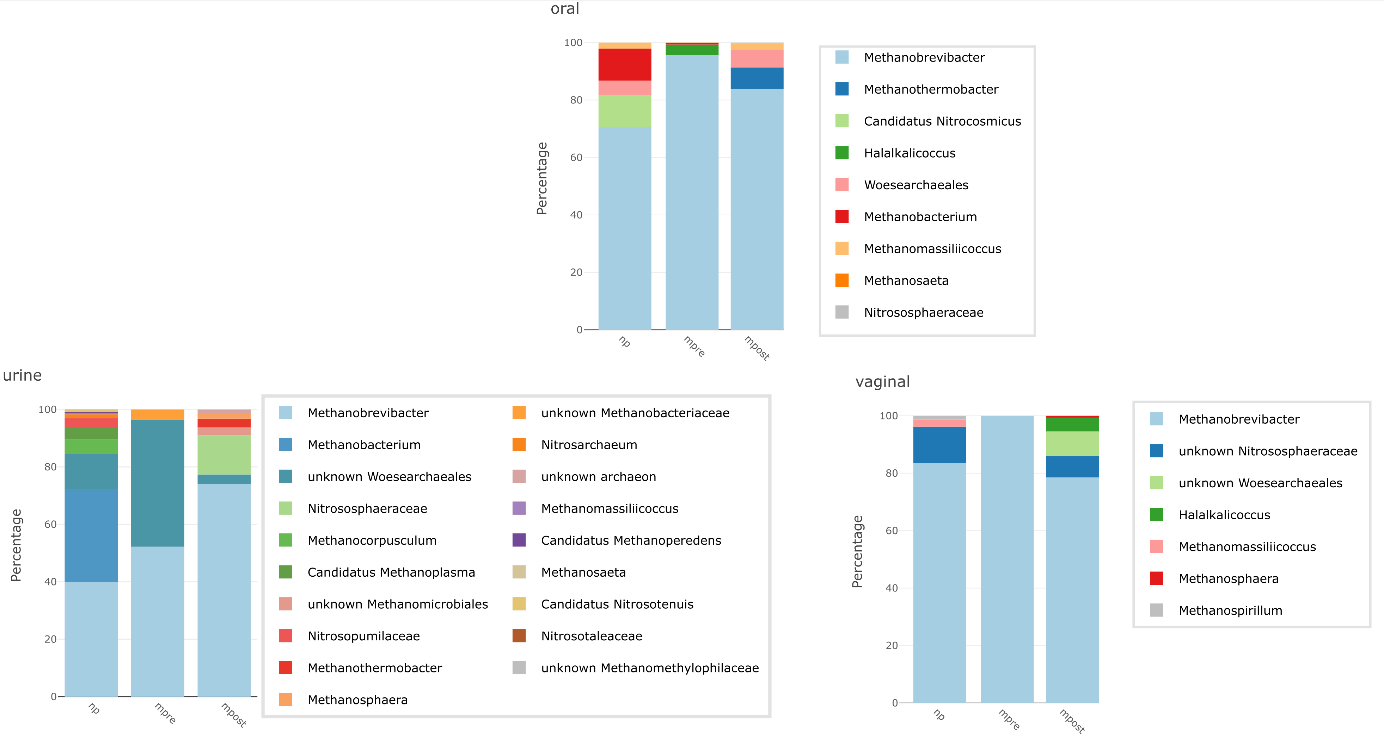


Suppl. Fig 9: stacked bar plots showing relative abundances of the topmost abundant archaeal genera depicted per group for oral, urine and vaginal samples.


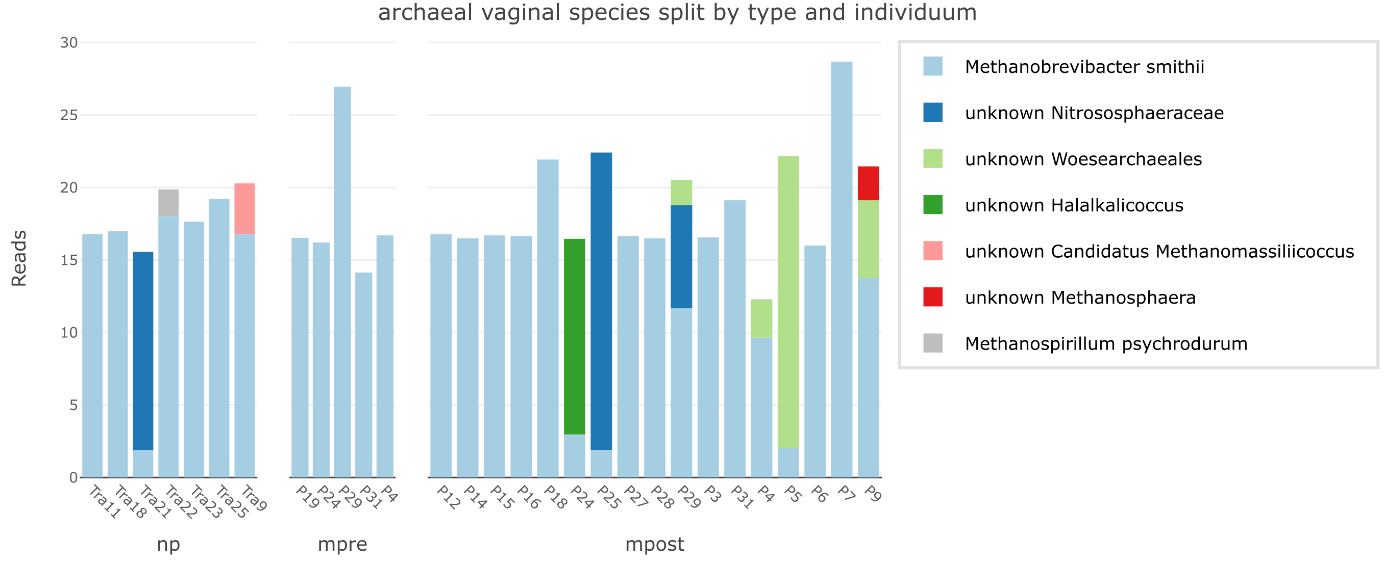


Suppl. Fig. 10: occurrence (reads, archaea nested PCR approach) of archaeal species in the vaginal microbiome, split by group per individuum.


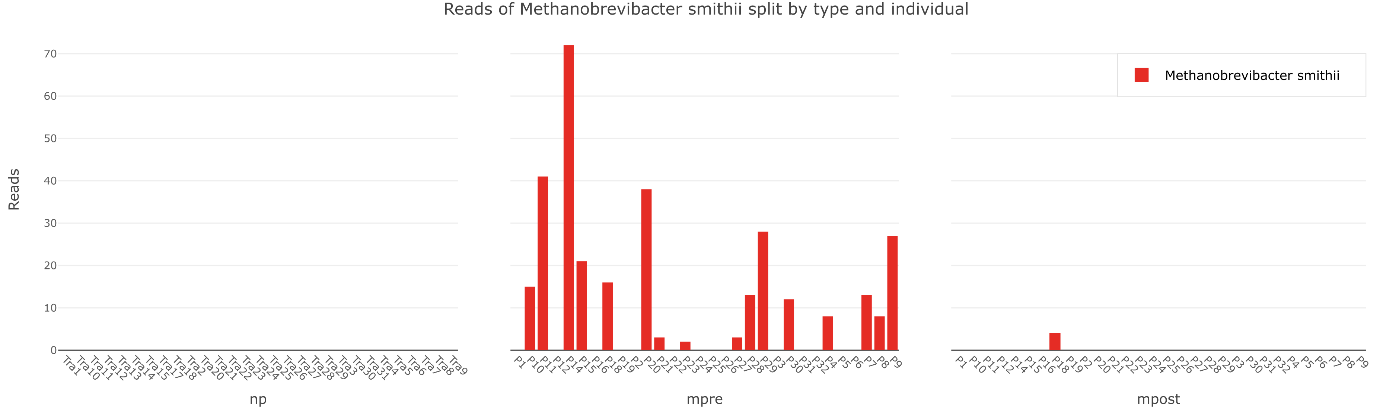


Suppl. Fig. 11: occurrence (reads, “universal” primers amplicon approach) of *M. smithii* in the oral microbiome, split by type per individuum


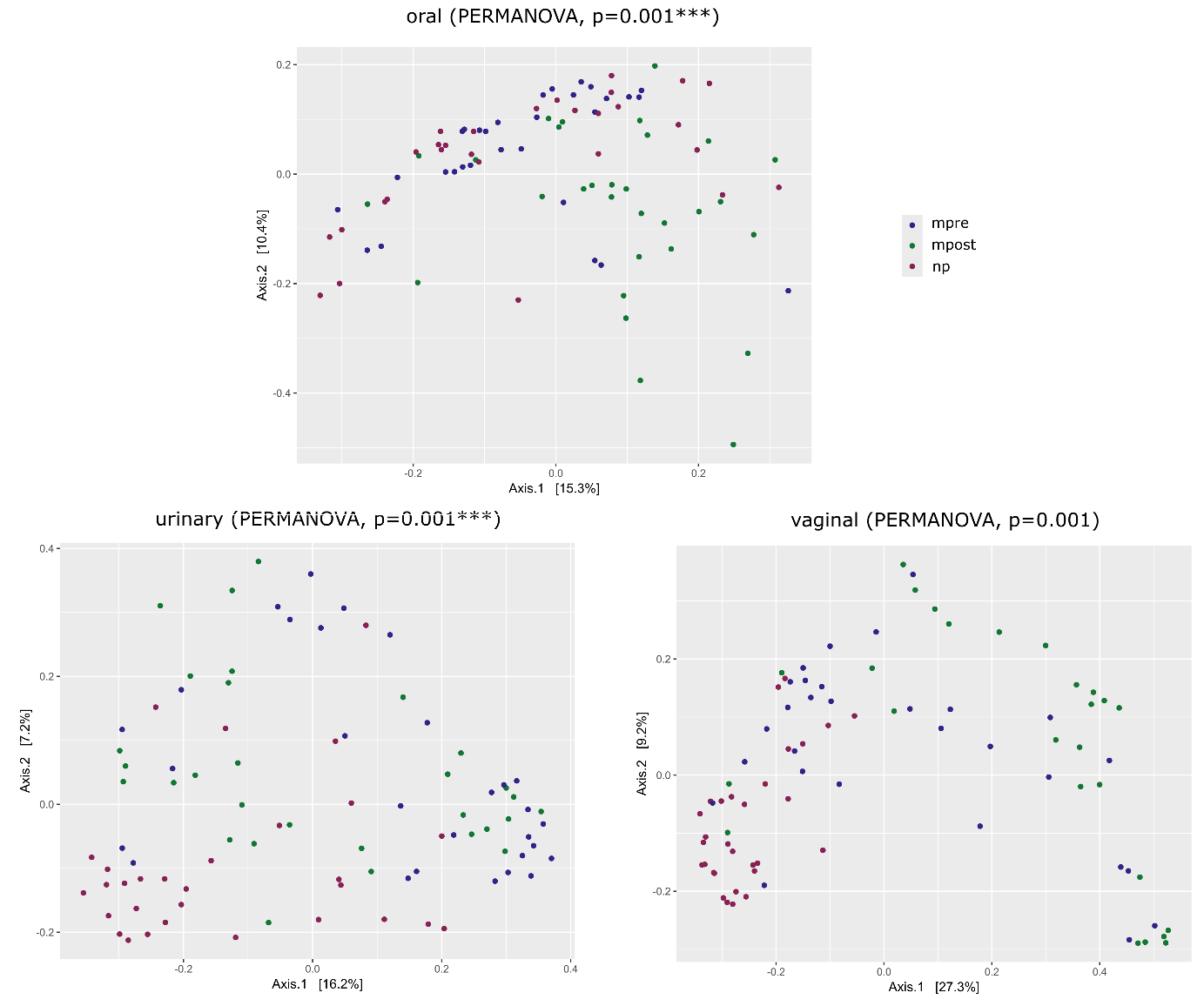


Suppl. Fig. 12: Principal Coordinate Analysis (PCoA) for groups (np, mpre, mpost) for bacterial ASVs with Unweighted UniFrac as distance Matrix and p-values, PERMANOVA


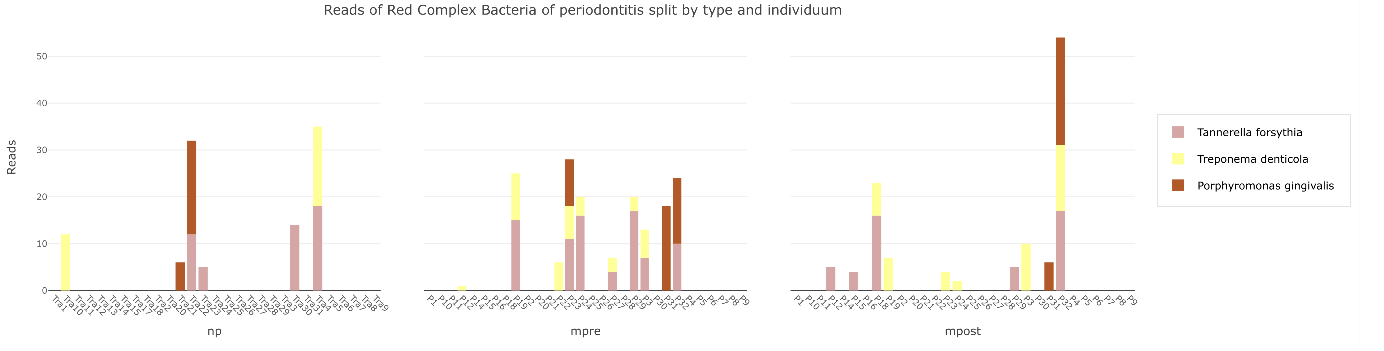


Suppl. Fig. 13: read numbers of red complex bacteria in the oral microbiome


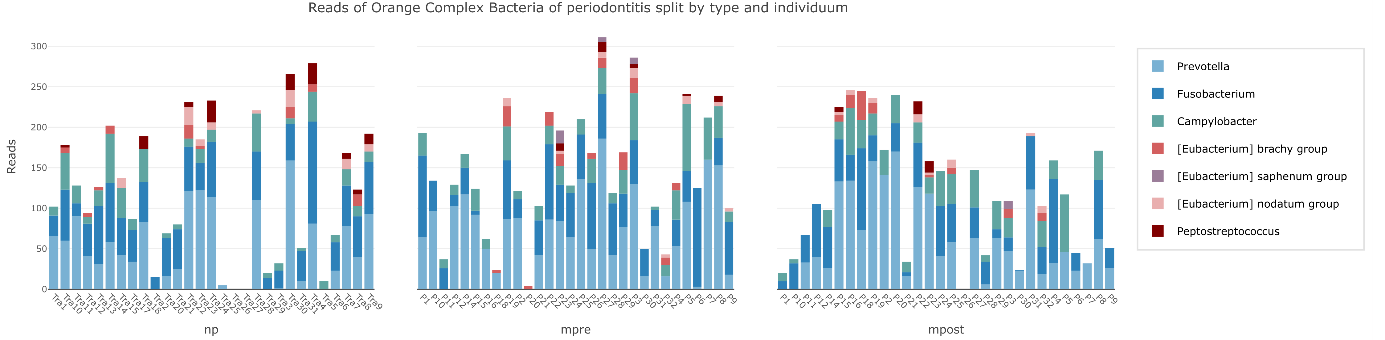


Suppl. Fig. 14: read numbers of orange complex bacteria in the oral microbiome


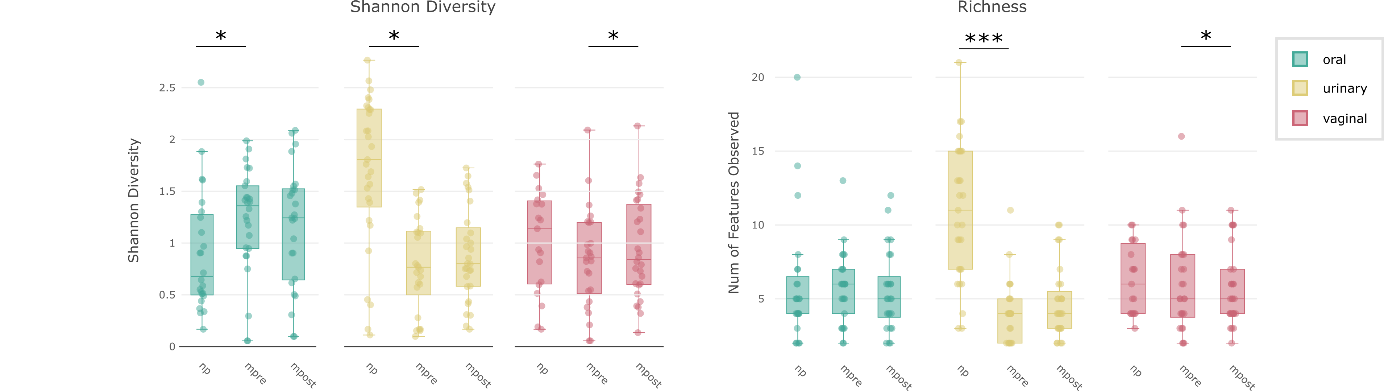


Suppl. Fig. 15: alpha diversity of the fungal microbiome on ASV level: Shannon diversity and richness, depicted per body site and split by group *p* < 0.05*, *p* < 0.005**, *p* < 0.001***


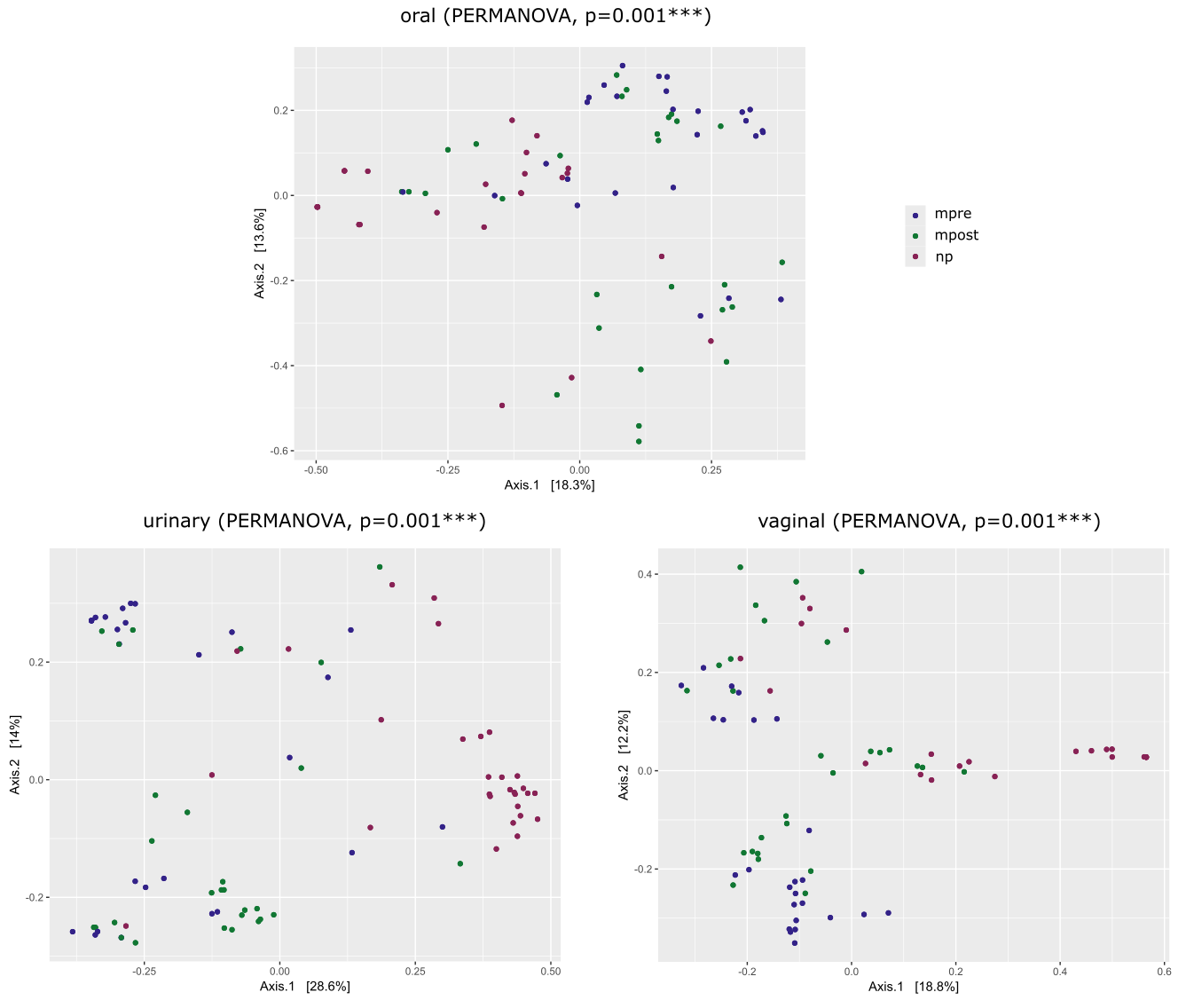
 Suppl. Fig. 16: Principal Coordinate Analysis (PCoA) for groups (np, mpre, mpost) for fungal ASVs with Unweighted UniFrac as distance Matrix and p-values, PERMANOVA


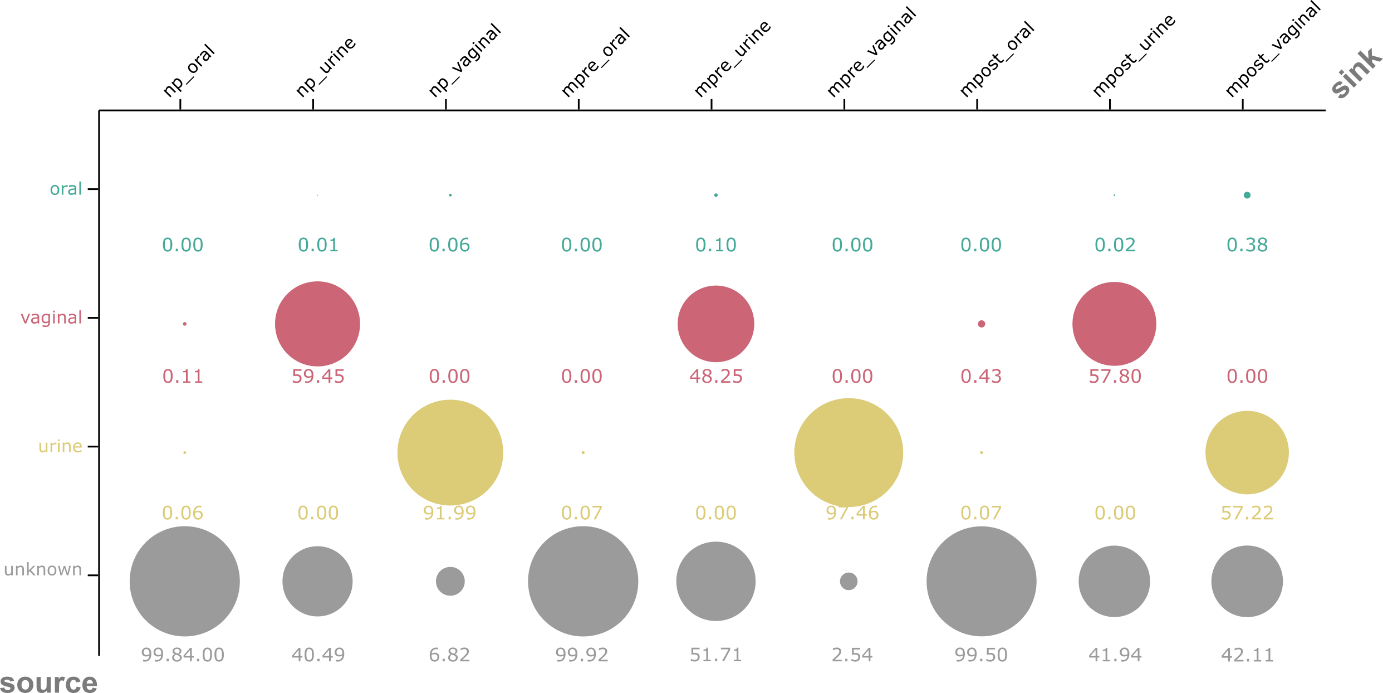


Suppl. Fig. 17: source tracking; comparison of oral, vaginal and urine samples with respect to their contribution from oral, urine, vaginal and unknown sources; dot sizes represent the proportion of contribution and numbers are given in percentage.


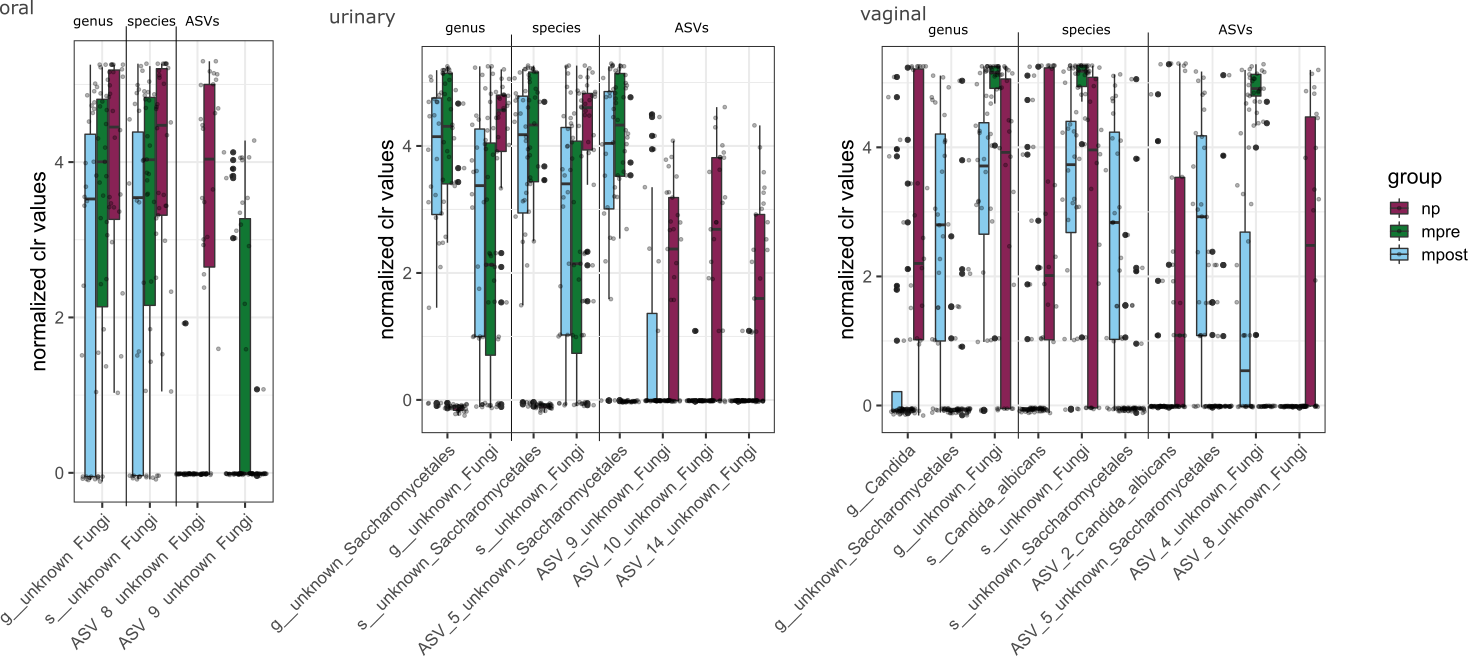
 Suppl. Fig. 18: differentially abundance boxplots of CLR transformed values on fungal genera (Aldex2) for oral, urine and vaginal samples.
